# Manipulating autophagic degradation in human diseases: from mechanisms to interventions

**DOI:** 10.1093/lifemedi/lnac043

**Published:** 2022-10-11

**Authors:** Yiqing Zhang, Xiaoxia Liu, Daniel J Klionsky, Boxun Lu, Qing Zhong

**Affiliations:** Key Laboratory of Cell Differentiation and Apoptosis of Chinese Ministry of Education, Department of Pathophysiology, Shanghai Jiao Tong University School of Medicine (SJTU-SM), Shanghai 20025, China; Key Laboratory of Cell Differentiation and Apoptosis of Chinese Ministry of Education, Department of Pathophysiology, Shanghai Jiao Tong University School of Medicine (SJTU-SM), Shanghai 20025, China; Department of Molecular, Cellular, and Developmental Biology, and the Life Sciences Institute, University of Michigan, Ann Arbor, MI 48109-2216, USA; State Key Laboratory of Medical Neurobiology and MOE Frontiers Center for Brain Science, Huashan Hospital, School of Life Sciences, Fudan University, Shanghai 200433, China; Key Laboratory of Cell Differentiation and Apoptosis of Chinese Ministry of Education, Department of Pathophysiology, Shanghai Jiao Tong University School of Medicine (SJTU-SM), Shanghai 20025, China

**Keywords:** design strategy, selective autophagy, targeted degradation

## Abstract

Targeted degradation, having emerged as a powerful and promising strategy in drug discovery in the past two decades, has provided a solution for many once undruggable targets involved in various diseases. While earlier targeted degradation tools, as exemplified by PROteolysis-TArgeting Chimera (PROTAC), focused on harnessing the ubiquitin-proteasome system, novel approaches that aim to utilize autophagy, a potent, lysosome-dependent degradation pathway, have also surfaced recently as promising modalities. In this review, we first introduce the mechanisms that establish selectivity in autophagy, which provides the rationales for autophagy-based targeted degradation; we also provide an overview on the panoply of cellular machinery involved in this process, an arsenal that could be potentially harnessed. On this basis, we propose four strategies for designing autophagy-based targeted degraders, including Tagging Targets, Directly Engaging Targets, Initiating Autophagy at Targets, and Phagophore-Tethering to Targets. We introduce the current frontiers in this field, including AUtophagy-TArgeting Chimera (AUTAC), Targeted Protein Autophagy (TPA), AUTOphagy-TArgeting Chimera (AUTOTAC, not to be confused with AUTAC), AuTophagosome TEthering Compound (ATTEC), and other experimental approaches as case studies for each strategy. Finally, we put forward a workflow for generating autophagy-based degraders and some important questions that may guide and inspire the process.

## Introduction

The majority of currently used drugs are inhibitors that utilize an occupancy-driven mode of action, in which the functional inhibition of target protein requires consistent binding, or occupancy, of the active site by drug compounds with high affinity [1, 2]. Most targets applicable for this mode of action fall into the categories of enzymes, ion channels and receptors, which possess well-defined active sites for high affinity inhibitor binding [3, 4]. However, these targets only cover <25% of known proteins of therapeutic interest, while the rest and the majority of disease-related targets, including many transcription factors, scaffold proteins, misfolded proteins, and other nonenzymatic proteins, are left undruggable [5]. These targets are involved in a wide spectrum of diseases including cancer, neurodegenerative diseases, and many other yet-unsolved clinical conditions, which leaves a significant vacancy without effective targeting methodologies [6].

This vacancy has been excitingly filled by emerging tools of targeted degradation, hijacking, and redirecting the cell-intrinsic quality control system by inducing proximity between a degradative cellular machinery and a desired target, as exemplified by PROteolysis TArgeting Chimera (PROTAC) and its variants [4, 7–11]. PROTAC is a type of bivalent compound consisting of a target ligand and an E3 ubiquitin ligase ligand, connected by a linker, which enables formation of a target-PROTAC-E3 ternary complex and subsequent target ubiquitination by the E3 [1, 2, 12, 13]. The ubiquitinated target is then degraded by the proteasome [14]. PROTACs are promising therapeutic modalities as several advantages of these degraders over traditional inhibitors have been observed, including an expanded target range covering many once-undruggable targets [2, 15, 16], the ability to silence both enzymatic and nonenzymatic functions [17–19], catalytic degradation and sustained effects [20–22].

Yet, PROTAC may not be a solution for all. While promising cases exist [23, 24], proteasomes may not be the optimal degradation route for protein aggregates related to Alzheimer’s Disease (AD), Huntington’s Disease (HD), Parkinson’s Disease (PD), and other neurodegenerative disorders, as many of them reportedly inhibit proteasomes, are proteasome-resistant, or produce cytotoxic products when degraded by proteasomes [25–29]; Degradation of bulky targets by proteasomes may also be inefficient [30]; Targets containing nonprotein materials—usually bulky as well—are intrinsically difficult to be completely digested by proteasomes. These targets are involved in a spectrum of human diseases, such as the defective mitochondria in Down syndrome [31, 32] and other neurological disorders [33], excessive lipid droplets in nonalcoholic fatty liver disease (NAFLD) or nonalcoholic steatohepatitis (NASH) [34], intracellular bacteria including *Streptococcus* [35], *Mycobacterium* [36, 37] among others [38], as well as viruses. Therefore, modalities that harness another major cellular quality control machinery, lysosomes, have been receiving increasing attention to complement proteasome-dependent targeted degradation tools [39–44].

Lysosomes harbor >60 hydrolytic enzymes capable of degrading a wider variety of substrates than proteasomes, including not only proteins, but also saccharides, lipids, nucleic acids, and other substances [45]. Substrates can be delivered to lysosomes via pathways including endocytosis, which degrades substrates from the extracellular space and plasma membrane, and autophagy, which degrades intracellular materials [43]. The endocytosis pathway has been exploited by modalities such as LYsosome TArgeting Chimera (LYTAC), Molecular Degraders of Extracellular proteins through the asialoglycoprotein receptor ASGR (MoDE-A) and Antibody-based PROTAC (AbTAC), as discussed elsewhere [39, 41, 46–49]. Meanwhile, autophagy also gained special attention and interest from developers of targeted degradation tools [39–41, 50]. Among the major forms of autophagy: macroautophagy, chaperone-mediated autophagy, and microautophagy, macroautophagy has been the most attractive route for targeted degradation due to its vast capability of degrading cargoes that are bulky or complex in molecular nature [51, 52]. Multiple approaches aiming at degrading targets via macroautophagy (hereafter autophagy) have been reported, as exemplified by AUtophagy-TArgeting Chimera (AUTAC), Targeted Protein Autophagy (TPA), AUTOphagy-TArgeting Chimera (AUTOTAC; not to be confused with AUTAC), and AuTophagosome TEthering Compound (ATTEC) [32, 53–57]; These autophagy-based bivalent degraders, termed MacroAutophagy Degradation Targeting Chimera (MADTAC) by Alabi and Crews [41], together with other experimental approaches of autophagy-based targeted degradation, have offered novel opportunities for both therapeutic and research applications.

However, despite rapid progress, what we have harnessed is considerably limited compared with the full potential of autophagy, leaving many untouched opportunities. To further exploit the autophagy-lysosome system for targeted degradation, this review first introduces the cellular machinery and mechanisms that drive autophagy and establish selectivity in autophagy. With these as background, we turn to the design strategies for autophagy-based degraders, with AUTAC, TPA, AUTOTAC, ATTEC, and other reported approaches presented as case studies in corresponding sections. Last, we present future perspectives on the development of novel autophagy-based degraders.

## Autophagy and selective autophagy

**Figure 1.**
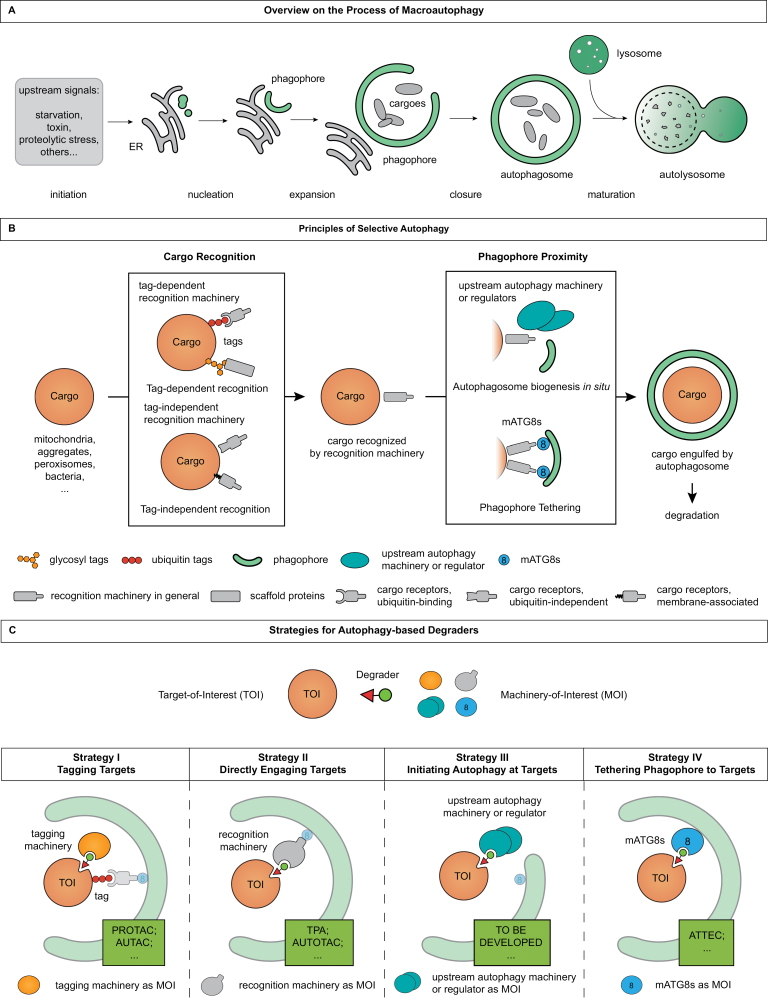
Autophagy and selective autophagy. (A) The process of autophagic degradation. Upon autophagy initiation, a membranous precursor to an autophagosome, termed a phagophore, is generated at specific sites on the ER in a nucleation process. Expansion and bending of the phagophore engulfs a portion of cytoplasm, referred as cargoes, and closure of the phagophore produces an autophagosome with double membranes. Finally, the autophagosome fuses with lysosomes to degrade cargoes together with its inner membrane. (B) Selective autophagy is mediated by a variety of recognition machinery that typically possess two basic functions: cargo recognition, and phagophore proximity, to enable spatiotemporal proximity between the cargo and the growing phagophore for selective engulfment. To recognize cargoes, a recognition machinery may bind to tags like ubiquitin or glycans that are added to or exposed from cargoes, or may directly bind to specific cargoes. For phagophore proximity, a recognition machinery may recruit and activate upstream autophagy machinery or regulators to the site of cargoes, which may promote autophagosome biogenesis *in situ*; this machinery may also tether the cargo in close proximity to a phagophore by binding to phagophore-coating mATG8s. Altogether, these mechanisms drive cargo engulfment by a phagophore for degradation. (C) Based on the mechanisms of selective autophagy, four strategies for autophagy-based degraders emerge according to the machinery-of-interest (MOI) they harness. Strategy I, exemplified by PROTACs and AUTACs, induces proximity between targets-of-interest (TOIs) and tagging machinery to add autophagy-prone tags to TOIs, which can be then recognized; strategy II, such as TPA and AUTOTACs, directly recruits recognition machinery to TOIs; strategy III utilizes upstream autophagy machinery or regulators as MOIs to trigger autophagosome biogenesis at the site of TOIs; strategy IV, as represented by ATTECs, brings TOIs and mATG8s together to tether TOIs to a phagophore.

The primary morphological feature of autophagy is the formation of autophagosomes, double-membraned vesicles containing cellular materials referred to as cargoes [52]. Following upstream signals that initiate autophagy, phagophore is generated at specific ER subdomains in a process termed nucleation, which then expands and bends to engulf a portion of cytoplasm; closure of the phagophore gives rise to a double-membraned autophagosome, which eventually fuses with lysosomes, resulting in the formation of an autolysosome, to degrade cargoes and its inner membrane, as comprehensively reviewed elsewhere (Fig. 1A) [58–63]. The process of autophagosome biogenesis is driven by a series of core autophagy machinery (hereafter autophagy machinery): The ULK1 complex, the class III phosphatidylinositol 3-kinase complex 1 (PtdIns3K-C1), WIPI proteins, ATG2-ATG9, and two ubiquitin-like conjugation systems, the ATG12-ATG5 conjugation system and the mammalian Atg8-family proteins (mATG8s)-phosphatidylethanolamine (PE) conjugation system (see Table 1), which function in an organized and hierarchical manner, along with other participants [51, 61, 64–66]. For detailed discussions, see various reviews [58, 59, 63, 67–70].

**Table 1.** Cellular machinery involved in selective autophagy

Group	Brief	Subgroup	Examples	Ref
Core Autophagy Machinery	Core autophagy machinery (hereafter autophagy machinery) are proteins that drive autophagosome biogenesis, building the autophagosome. In selective autophagy, autophagy machinery can be recruited to the cargo.	ULK1 Complex	ULK1*, FIP200*, ATG13, ATG101	[58, 63, 67, 69]
		PtdIns3K-C1	VPS34, VPS15, ATG14L, Beclin-1, NRBF2	[58, 63, 67, 69]
		WIPIs	WIPI1, WIPI2, WIPI3, WIPI4	[63, 69]
		ATG2s-ATG9	ATG2A, ATG2B; ATG9A	[58, 63, 67, 69, 71]
		ATG12–ATG5 Conjugation System	ATG7, ATG10, ATG12, ATG5	[72]
		mATG8–PE Conjugation System	ATG4, ATG7, ATG3, ATG12-ATG5-ATG16L1; mATG8s (LC3A, LC3B*, LC3B2, LC3C, GABARAP, GABARAPL1, GABARAPL2)	[72–74]
Cargo Receptors	Cargo receptors are molecular tethers whose major function is to associate cargoes with a phagophore. For this purpose, cargo receptors interact with their cargoes (binding to tags or to cargoes directly) and phagophore components (in most cases, mATG8s) simultaneously. Some cargo receptors are also capable of recruiting autophagy machinery to promote local autophagosome biogenesis	Soluble, Ubiquitin-dependent Cargo Receptors	p62*, NBR1, NDP52*, TAX1BP1, OPTN*, TOLLIP, CCDC50	[75, 76]
		Soluble, Ubiquitin-independent Cargo Receptors	NCOA4, STBD1, NUFIP1, AMBRA1*, TRIM5, TRIM20, TRIM21, CCT2	[77–81]
		Membrane-associated Cargo Receptors	BNIP3, NIX, FUNDC1, BCL2L13, FKBP8, PHB2, Cardiolipin, Ceramide; CCPG1, TEX264, SEC62, ATL3, FAM134B, RTN3L, CALCOCO1, GOLPH3, PEX14	[82–90]
Scaffold proteins	Scaffold proteins basically coordinates cargoes, cargo receptors, autophagy machinery, and their regulators to promote their assembly and activation	N/A	ALFY, Huntingtin, UXT, WDR81, TRIM13, TRIM16, Galectin-3, Galectin-8, Galectin-9, NIPSNAP1, NIPSNAP2	[91–101]
Tags and Tagging Machinery	Some cargo receptors or scaffold proteins bind to tags rather than to cargoes directly. Tags could be added or exposed from cargoes	Ubiquitin Tagging	Ubiquitin*, various E3 ligases including Parkin (downstream of PINK1*), CHIP*, LRSAM1, and others	[102–104]
		Glycan Tagging	Glycans (exposed from damaged endosomes or lysosomes)	[65, 105, 106]
		N-degron Tagging	N-terminal Arg* (Nt-Arg, which can be added to protein N terminus by ATE1), and others	[98, 107–109]
Other Regulators	Some regulators could be recruited to the site of cargoes to promote local activity of autophagy machinery, cargo receptors, and other cellular machinery	N/A	TBK1*, AMPK, and others	[110–113]

Abbreviations: AMBRA1, autophagy and Beclin-1 regulator 1; AMPK, AMP-activated kinase; ATE1, arginyltransferase 1; ATG, autophagy-related; ATL3, atlastin GTPase 3; BCL2L13, BCL2 like 13; NIX, NIP3-like protein X; CALCOCO1, calcium binding and coiled-coil domain 1; NDP52, nuclear dot protein 52 kDa; CCDC50, coiled-coil domain containing 50; CCPG1, cell cycle progression protein 1; FKBP8, FKBP prolyl isomerase 8; FUNDC1, FUN14 domain containing 1; GABARAP, GABA type A receptor-associated protein; GOLPH3, Golgi phosphoprotein 3; LRSAM1, leucine rich repeat and sterile alpha motif containing 1; LC3, microtubule associated protein 1 light chain 3; NBR1, NBR1 autophagy cargo receptor; NCOA4, nuclear receptor coactivator 4; NIPSNAP, nipsnap homolog; NRBF2, nuclear receptor binding factor 2; NUFIP1, nuclear FMR interacting protein 1; OPTN, optineurin; PEX14, peroxisomal biogenesis factor 14; PHB2, prohibitin 2; PINK1, PTEN induced kinase 1; PtdIns3K-C1, class III phosphatidylinositol 3-kinase complex 1; FAM134B, family with sequence similarity 134, member B; RTN3L, reticulon 3 long isoform; FIP200, FAK family kinase-interacting protein of 200 kDa; SQSTM1/p62, sequestosome 1; STBD1, starch binding domain 1; CHIP, Carboxy terminus of Hsp70-interacting protein; TAX1BP1, Tax1 binding protein 1; TBK1, TANK binding kinase 1; TEX264, testis expressed 264, ER-phagy receptor; TOLLIP, toll interacting protein; TRIM, tripartite motif containing; ULK1, unc-51 like autophagy activating kinase 1; UXT, ubiquitously expressed prefoldin like chaperone; ALFY, Autophagy-linked FYVE protein; WDR81, WD repeat domain 81; WIPI, WD repeat domain, phosphoinositide interacting.

*Asterisks indicate that the cellular machinery has been utilized for a targeted degradation purpose in proof-of-concept experiments or as a *bona fide* degrader.

Autophagy was initially discovered as a non-selective degradation pathway in response to starvation and other stresses [114]. However, it was later revealed that autophagic degradation of many cargoes, including mitochondria, peroxisomes, ER, and cytosolic pathogens, can be selective [115, 116]. Therefore, autophagy can be classified into two main types: bulk autophagy, where the phagophore may randomly engulf nearby cellular contents [59]; and selective autophagy, where the process of autophagosome biogenesis can be spatiotemporally coordinated in proximity with specific cargoes for selective engulfment and degradation [75, 117].

The key to selectivity lies in the panoply of cellular machinery that establishes proximity between the cargo and the phagophore, most of which fall into categories of cargo receptors, also known as cargo adaptors [73, 76], and scaffold proteins [118], collectively termed recognition machinery in this review. In brief, to bridge between cargo and phagophore, a typical recognition machinery should possess at least two basic activities: cargo recognition, and phagophore proximity [76, 118], as shown in Fig. 1B. Cargo recognition is the process that identifies cargo from other cellular materials [73, 119, 120]. In some cases, this requires cargo tagging, in which recognition machinery binds to tags or “eat-me” signals that are added to or exposed from a cargo. The best characterized tag to date is ubiquitin, while other tags, such as glycans, are also involved in marking specific cargoes [65, 120–122]. In other cases, the recognition machinery can specifically and directly bind to their cargoes, or are incorporated into cargoes like ER or mitochondria as a membrane component [76, 121]. As for phagophore proximity, while earlier studies have focused on cargo receptors as molecular tethers that simultaneously interact with cargoes and phagophore-coating mATG8s, an updated model emphasizes the participation of upstream autophagy machinery such as the ULK1 complex, which can be recruited to cargo and may drive autophagosome biogenesis spatiotemporally adjacent to the cargo [59, 73, 75, 76, 117]. The latter process, termed “cargo-induced autophagy,” “precision autophagy,” “phagophore formation *in situ,*” or “on demand autophagosome biogenesis” [59, 76, 91, 110, 123] elsewhere, are collectively referred to as autophagosome biogenesis *in situ* in this review.

From the above discussion, it becomes apparent that selective autophagy and autophagy-based targeted degradation share a common basis, as both processes would induce proximity between the substrate and the phagophore. Thus, understanding the mechanisms of selective autophagy will provide great insights into development of autophagy-based degraders [40]. In the following sections, we briefly introduce how selective autophagy is driven by the cooperation of the aforementioned variety of cellular machinery.

### Tag-dependent recognition

#### Ubiquitin

Ubiquitination has been the most studied tagging mechanism in selective autophagy [75, 121, 122]. During ubiquitination, the C-terminal Gly residue of ubiquitin is covalently attached to Lys (alternatively Met or Cys) residues on the substrate under the catalytic action of a series of enzymes: E1 activating enzymes, E2 conjugating enzymes, and E3 ligases, among which E3s directly interact with substrates and transfer a ubiquitin moiety to the substrate [14, 122, 124]. Ubiquitin tags are heterogeneous in structure as they can contain variable numbers of ubiquitin moieties (from monoubiquitin to polyubiquitin), have different linkage types between two adjacent ubiquitin moieties (such as K48- and K63-) or even branch out [125, 126]. This structural complexity endows ubiquitination with diverse functional outcomes including proteasomal degradation, autophagy, and many others [28, 125, 127, 128].

Reported autophagic cargoes that can be recognized via ubiquitination include misfolded proteins and their aggregates [129, 130], mitochondria 82,102,131–134], peroxisomes [103, 135], several species of cytosolic bacteria [104,136–142], ER [98], proteasomes [143, 144], and others, as summarized elsewhere [75, 120]. Ubiquitination of these cargoes requires recruitment and activation of corresponding E3s, which result from a variety of stimuli and pathways (such as the PINK1-Parkin pathway for mitophagy). These pathways are beyond our scope and thus will not be discussed in detail. Ubiquitinated cargoes are then recognized by a variety of recognition machinery, including ubiquitin-binding cargo receptors like SQSTM1/p62, NBR1, CALCOCO2/NDP52, TAX1BP1, OPTN, TOLLIP, and the recently identified CCDC50 [76, 145], together with scaffold proteins like WDFY3/ALFY [92,93,146], Huntingtin [94], WDR81 [147], UXT [148], and some TRIM family proteins [95, 149] (summarized in Table 1). They cooperate to direct ubiquitinated cargoes to autophagic removal.

The fact that cargoes can be directed to autophagic degradation via ubiquitination indicates that inducing target ubiquitination is a possible way to direct the target to autophagic destruction. Indeed, such attempts lead to autophagic degradation in some, but not all, reports. To better harness ubiquitination by autophagy-based targeted degraders, it is necessary to understand how ubiquitinated cargoes can be directed to autophagy rather than other routes—for instance, proteasomal proteolysis [40, 128]. In other words, does a type of autophagy-prone ubiquitin tag exist?

It is true that properties of ubiquitin tags affect their pathway preference. Proteins carrying longer polyubiquitin chains or multiple polyubiquitin chains are more readily captured by oligomeric p62 to form phase-separated condensates, which favors autophagic over proteasomal degradation [150–154]. However, the requirement on ubiquitin chain may vary according to the cargo or recognition machinery, as shorter ubiquitin chains seem more effective than longer ones in recruiting OPTN for mitophagy [77]. A certain extent of preference of linkage type likely also exists, as K63- or M1-linked (linear) polyubiquitin chains are more potently captured by p62 oligomers and OPTN than K48-linked chains [152, 155–158], while K48- or K11-linked polyubiquitin are readily recognized by proteasomes [159, 160]. K63-polyubiquitination of cytosol-invading *Streptococcus*, triggered by S-guanylation of bacterial proteins, is also necessary for autophagic clearance of the bacteria [137].

However, these lines of evidence are mostly suggestive and no definitive autophagy-inducing ubiquitin tag can be concluded to date. Meanwhile, it is also important to note that properties of ubiquitin tags per se may not be the only factor affecting autophagic recognition. For example, mitochondrial fission is required for effective ubiquitin-mediated mitophagy, which is largely disrupted in fission-defective cells despite the fact that mitochondrial ubiquitination is unaffected [161, 162].

#### Glycans

Glycans have been reported to be another important degradative tag apart from ubiquitin, especially for damaged endosomes and lysosomes [65]. In these membranous compartments, lumen-facing proteins are highly glycosylated, while cytosol-facing proteins are not [163]. Upon membrane rupture, glycoproteins can be exposed to the cytosol, where they are recognized to trigger membrane repair or autophagic removal [105, 164]. We only discuss autophagy-related consequences of glycan exposure in this review, and for some discussion on how the choice between repair and removal is made, we direct readers to some recent reviews [105, 106]. In addition to these compartments, some species of cytosol-invading bacteria, such as *Streptococcus*, also express surface glycans that could be recognized by selective autophagy [138].

On the one hand, exposure of glycan tags can recruit sugar-binding E3s, such as SCF ^FBXO27^ and SCF ^FBXO2^ from the SCF (Skp1, Cullin, F-box containing) family, to ubiquitinate the target, which leads to ubiquitin-mediated recognition [138, 165–168]. On the other hand, exposed glycan tags can also be directly recognized by cytosolic Galectins, a family of β-galactoside-binding lectins [105, 169]. Reported Galectins that are involved in autophagic recognition of glycans are Galectin-3 and Galectin-8. These proteins are multifunctional scaffolds that can recruit a variety of effectors, including E3s such as Parkin [170], cargo receptors like NDP52 and TAX1BP1 [96,171,172], other scaffold proteins such as TRIM16 [97], and various autophagy machinery [97]. Galectins thus provide a bridge between glycan exposure and autophagic removal.

#### N-degrons

N-degrons are amino acid residues at the N terminus of proteins conventionally viewed as proteasomal-targeting tags that regulate the half-life of proteins [107]. While N-degrons are commonly recognized by E3s for subsequent proteasomal proteolysis [161], some N-degrons including Nt-Arg and others can be recognized by at least one cargo receptor, p62, for autophagic degradation of proteins, which was reported to be more prominent during proteolytic stress (Table 1) [107, 173]. Known cargoes that are degraded in this way include HSPA5/BiP, CALR (calreticulin) and PDI (protein disulfate isomerase), which are ER-lumen chaperones that are exposed to the cytosol before arginylated at their N-terminus by ATE1 (arginyltransferase 1) [108]. Degradation of these chaperone proteins along with their misfolded clients and ER portions may alleviate ER stress [98]. Importantly, upon binding to the ZZ domain of p62, Nt-Arg also induces a conformation change of p62 that promotes p62 oligomerization and cargo recognition, which might also enhance its recognition of ubiquitinated cargoes [98, 108, 109, 174].

### Tag-independent recognition

While phagophore targeting of some cargoes is mediated by tags, others are not. Such cargoes are targeted to a phagophore by recognition machinery that bind to cargoes directly; thus, unlike tag-dependent recognition machinery that could bind to a variety of cargoes as long as they are properly tagged, tag-independent recognition machinery usually only recognize specific cargoes [75, 76].

Many of this type of recognition machinery is anchored to membranous organelles via membrane-binding domains [76, 82, 175]. Some studied examples include CCPG1 [176–178], RETREG1/FAM134B [83,179–181], RTN3L [182, 183], TEX264 [184, 185], SEC62 [186], and ATL3 [187] for the ER; and BNIP3 [82], BNIP3L/NIX [82], FKBP8 [188], BCL2L13 [189–191], FUNDC1 [192], and PHB2 [193] for mitochondria (Table 1). In addition, cardiolipin and ceramide, two mitochondrial lipids that are exposed to the cytoplasm and bind to mATG8s in response to mitochondrial stress, can be also viewed as cargo receptors in this category [84,194,195]. Some other cases include the scaffold proteins NIPSNAP1 and NIPSNAP2, whose internalization into the mitochondrial lumen requires normal mitochondrial function. Thus, these proteins would accumulate on the surface of damaged mitochondria where they recruit p62, NDP52, and ALFY to mediate mitophagy [99].

Several other tag-independent cargo receptors are soluble. A well-studied example is NCOA4, a dimeric cargo receptor that binds to the FTH1 subunit of the ferritin particle [77, 196]. Because a ferritin particle contains 48 FTH1 subunits, multivalent interactions between dimeric NCOA4 and ferritin result in phase-separation and formation of ferritin-containing condensates [197]. It remains unknown whether other tag-independent cargo receptors are also capable of forming phase-separated condensates with their cargoes [78, 110, 111, 198–201]. In addition, ubiquitin-binding cargo receptors may also directly recognize certain cargoes in a tag-independent manner, as reported for p62 [202, 203], NBR1 [204, 205], NDP52 [206, 207], OPTN [208], and TOLLIP [209] (Table 1).

### Autophagosome biogenesis *in situ*

The ULK1 complex and PtdIns3K-C1 are considered as two major protein assemblies involved in autophagy initiation [67, 210]. Together with ATG9-containing vesicles, ATG16L1, and other effectors, are vital for forming the autophagosome precursor [63, 67, 211]. These upstream executors of autophagy can be recruited to cargoes, which may couple the early events of autophagosome biogenesis with the cargo [59, 123].

While tethering cargo to a phagophore by binding to mATG8s, some cargo receptors, including p62, NDP52, TAX1BP1, and CCPG1, are additionally capable of recruiting the ULK1 complex by interacting with RB1CC1/FIP200 [91, 123, 176, 212]. In addition, NBR1 and OPTN were shown to bind to FIP200 *in vitro* [177, 213]. Furthermore, both mitophagy receptors FUNDC1 [214] and BCL2L13 [191] can recruit ULK1, the latter of which is dependent on LC3B. Mitochondrial recruitment of the ULK1 complex is sufficient to trigger autophagic degradation of mitochondria, which bypasses mTORC1 or AMPK and can still occur in the absence of major ubiquitin-binding cargo receptors (p62, NBR1, TAX1BP1, NDP52, and OPTN) [112]; Recruitment of the ULK1 complex by TAX1BP1 to NBR1-containing condensates also mediates autophagic degradation even when mATG8 lipidation is abolished by *ATG7* knockout [215].

What occurs following ULK1 complex recruitment to the cargo is not understood well, but the complex is activated at the site of the cargo, likely by crowding and autophosphorylation, which may coordinate nucleation events with cargo [112, 123, 212]. The mitochondria-localized ULK1 complex associates with nearby ER sheets, which may provide a nucleation platform [134]; in addition, the ULK1 complex seems to oscillate on and off mitochondria several times before a portion of the mitochondria is engulfed by a phagophore, suggesting multiple nucleation events [216, 217]. Nucleation may be positively regulated by cargo receptors, as the membrane-binding activity of FIP200 is allosterically enhanced by NDP52, which may promote cargo-bound ULK1 complex interaction with the ER [218]. After nucleation, it has been suggested that FIP200 can be outcompeted by phagophore-coating mATG8s from p62 to prevent excessive degradation of the autophagy machinery [123, 212]. Still, more research will be needed to elucidate the process.

Cargo recruitment of the ULK1 complex can be positively regulated by TBK1 (TANK binding kinase 1), a Ser/Thr kinase that can be recruited by cargo receptors including NDP52 [91, 112, 219], TAX1BP1 [220], and OPTN [219, 221]. TBK1 is activated following its recruitment to cargoes, which is suggested to result from local clustering and auto-phosphorylation [112, 222–224]. Activated TBK1 promotes recruitment of FIP200 by NDP52 [112] and OPTN [177]. In addition, phosphorylation by TBK1 also increases the affinity of p62 [221, 225, 226], NDP52 [72], TAX1BP1 [221], and OPTN [158, 221, 227] to ubiquitinated cargoes or mATG8s, which promotes cargo recognition and phagophore tethering.

Apart from ULK1, other autophagy machinery can be recruited to cargo as well. An interesting class of cellular machinery involved in coordinating these autophagy machinery are TRIM family E3s [228]. While some TRIMs participate in autophagy by regulating autophagy machinery such as ULK1 and Beclin-1 by ubiquitination as E3s, or functioning as cargo receptors [97], some TRIMs behave as scaffold proteins capable of forming structures termed “TRIMosomes” that may contain autophagy machinery including ULK1, Beclin-1 or ATG16L1, cargo receptors including p62, and regulators such as AMPK [110, 201, 229]. Thus, they may promote assembly and activation of autophagy machinery at the site of recognized cargoes.

ATG9, another upstream autophagy machinery, can be recruited to cargo as well. In mitophagy, this requires OPTN, and the abolishment of ATG9-OPTN interaction leads to mitophagy failure [230]; in pexophagy, ATG9 recruitment involves PEX14 (peroxisomal biogenesis factor 14), a peroxisomal surface cargo receptor together with scaffold proteins TNKS (tankyrase) and TNKS2 (tankyrase 2) [231]. ATG12–ATG5-ATG16L1 can also be recruited by cargo receptors including p62, NDP52, TAX1BP1, and OPTN, and scaffold proteins such as ALFY and some TRIMs [92, 97, 110, 232–235]. It seems that cargo receptors can enhance the enzymatic activity of ATG12-ATG5-ATG16L1, as shown in an *in vitro* experiment using NDP52, TAX1BP1, and OPTN [234]. Recruitment of this E3-like enzyme complex is thought to promote phagophore assembly at the site of cargo [72, 233, 236]. However, whereas both ATG9 vesicles and ATG16L1 vesicles are implicated in nucleation, whether their recruitment to cargo is sufficient to trigger autophagosome biogenesis remains unknown.

### Phagophore tethering

Phagophore tethering results from interactions between cargo receptors and mATG8s, which coat the phagophore, allowing for engulfment of cargoes and exclusion of non-cargo materials [73, 76, 117]. Most cargo receptors interact with mATG8s via their LC3-interacting region (LIR), a conserved motif that docks into a LIR-docking site (LDS) on mATG8s. LIRs from different cargo receptors differ in structure and may exhibit diversities in affinity and binding preference towards individual mATG8s [73, 74]. Apart from the LIR, the ubiquitin-interacting motif (UIM) and motif interacting with ubiquitin (MIU) are two other mATG8-binding regions found in several cargo receptors, both of which dock into an UIM-docking site (UDS), a binding pocket that typically lies on the opposite side to the LDS [145, 237]. Furthermore, cardiolipin and ceramide also show atypical interactions with mATG8 [74, 194, 195]. Altogether, interactions between cargo receptors and mATG8s could provide insights into developing novel ligands for mATG8s, as seen in some recent work [238, 239].

As mentioned above, an interesting property of some cargo receptors is forming gel-like condensates with their cargoes via liquid–liquid phase separation (LLPS), which usually results from multivalent weak interactions between oligomeric cargo receptors and cargoes with multiple binding sites [150, 153–155, 240]. The most studied case in mammalian cells is seen with p62, which forms condensates with ubiquitinated proteins and is implicated in clearing aggregates and mitochondria, while a recent study found that NCOA4 also undergo LLPS with ferritin [150, 151, 197, 241–243]. Condensate formation contributes to autophagic clearance by increasing the local density of cargo receptors and facilitates their interactions with phagophore-coating mATG8s [151, 241], and may promote phagophore bending to engulf a portion of condensates via a “wetting” process [244].

## Strategies for autophagy-based degraders

Here, autophagy-based degraders are defined as bivalent compounds that induce close proximity between a Target-of-Interest (TOI) and a phagophore by recruiting a Machinery-of-Interest (MOI), a cellular machinery involved in selective autophagy, to trigger selective, autophagic removal of the TOI. The mode-of-action of an autophagy-based degrader would be determined by the behaviors of the MOI it harnesses, which, as discussed above, can be classified into four major groups, including tag-dependent and tag-independent recognition, autophagosome biogenesis *in situ*, and phagophore tethering. Thus, four corresponding strategies for designing autophagy-based degraders arise, as depicted in Fig. 1C:

Degraders using the first strategy, *Tagging Targets*, aim at modifying targets with autophagy-prone “eat-me” tags by hijacking endogenous tagging machinery such as E3s. This hijacking is expected to be followed by tag-dependent autophagic recognition and phagophore delivery. Successful examples for this strategy are AUTACs, which mimic S-guanylation to induce target K63-polyubiquitination and subsequent autophagic clearance [32].

Degraders using the second strategy, *Directly Engaging Targets*, directly recruit recognition machinery to targets without tagging the target. Successful cases of this strategy include the TPA technology, exemplified by two compounds (BMF1-64 and BMF-1-141) that recruit p62 to desired targets for autophagic degradation [54]; and AUTOTACs, which utilize an activating ligand of p62 to induce target coaggregation with p62 [53].

Degraders using the third strategy, *Initiating Autophagy at Targets*, recruit key autophagy machinery or their regulators to a target, which is expected to trigger autophagosome biogenesis *in situ*—at the site of the target. Successful examples for this strategy include some experimental approaches recruiting ULK1, TBK1, or other autophagy machinery to organelles for degradative effects [112].

Degraders using the fourth strategy, *Tethering Phagophore to Targets*, tether targets to a nearby phagophore by bridging between the target and mATG8s, which may facilitate target engulfment by the phagophore. This is exemplified by ATTECs [55–57].

### Strategy I: tagging targets

#### PROTAC

**Figure 2.**
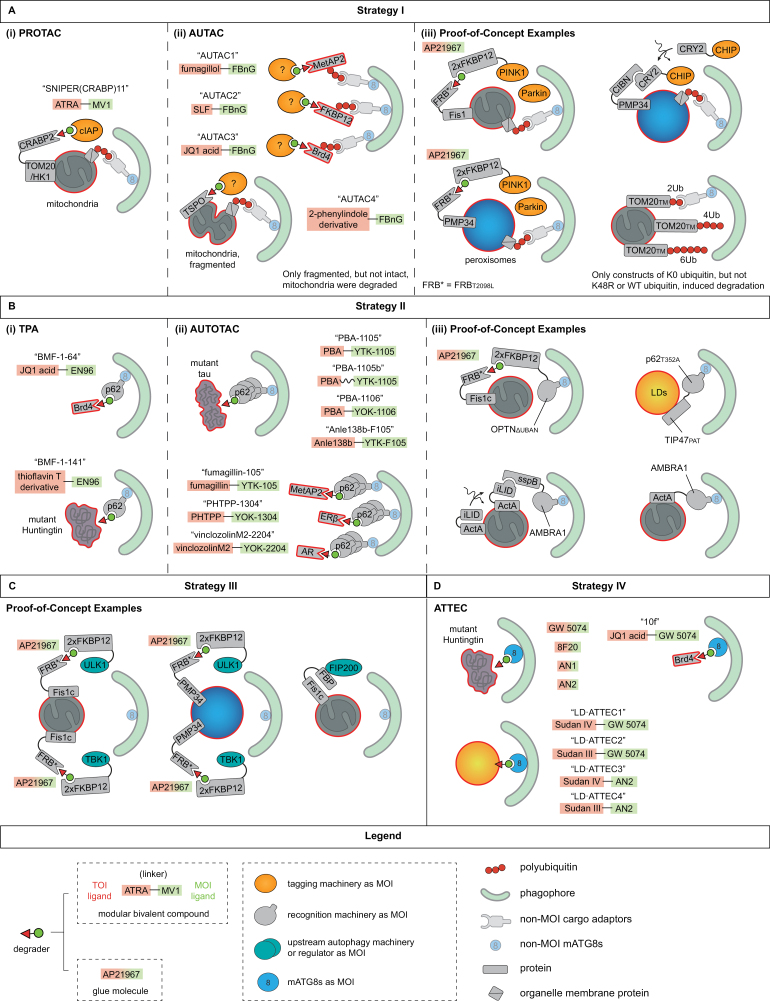
*Bona fide* autophagy-based degraders. (A) Approaches following Strategy I add autophagy-prone tags to TOIs. (i) A PROTAC, SNIPER(CRABP)11, recruits IAP family E3s to mitochondria surface proteins (CRABP2-tagged TOMM20-HK1) to induce mitochondrial degradation. (ii) AUTAC degraders succeeded in targeting METAP2, FKBP12, and BRD4 (a nucleus-localized protein) to autophagic degradation by inducing K63-polyubiquitination of these TOIs via an unknown E3. Moreover, another mitochondria-targeting AUTAC (binding to TSPO, a surface protein) lead to autophagic clearance of fragmented, but not intact, mitochondria. (iii) Other proof-of-concept experiments. PINK1_Δ1-110_-2xFKBP is recruited to mitochondria-localized FRB ^T2098L^-FIS1, or peroxisome-localized FRB ^T2098L^-PMP34, respectively. PINK1 is a kinase that can recruit and activate Parkin, an E3. In another approach, CHIP, an E3, was attached to a CRY2 moiety that undergoes a conformational change and dimerizes with peroxisome-localized CIBN-PMP34 upon blue light treatment. Ectopic expression of linear ubiquitin chains on mitochondria by attaching them to the transmembrane domain of TOMM20 also induces mitophagy. In this case, only ubiquitin chains consisting of K0, rather than K48R and WT, ubiquitin moieties succeed; shorter chains containing 2 ubiquitin moieties are more effective than longer ones. (B) Approaches following strategy II recruit recognition machinery to TOIs. (i) TPA technology utilizes bivalent degraders that covalently bind to p62, a cargo receptor, which succeeds in degrading BRD4 and mutant Huntingtin via autophagy. (ii) AUTOTAC also utilizes p62 as part of the MOI. Apart from recruiting p62 to TOIs, AUTOTAC degraders induce oligomerization of p62, which activates the cargo receptor for mediating autophagy. AUTOTAC has succeeded in a variety of TOIs including both aggregating and soluble proteins. (iii) Other proof-of-concept approaches. In one system, 2xFKBP-OPTN dimerizes with mitochondria-localized FRB ^T2098L^-FIS1c (C terminus of FIS1) upon treatment with AP21967. Another system utilizes mitochondria-localized iLID-ActA, which undergoes a conformation change and recruits sspB-AMBRA1 under blue light. Ectopically expressed cargo receptors, such as TIP47[PAT]-p62 ^T352A^ (localized to LDs) and AMBRA1-ActA (localized to mitochondria), also yield degradative outcomes. (C) Approaches following strategy III aim to recruit upstream autophagy machinery or regulators as MOIs to TOIs. (i) ULK1 or TBK1, attached to 2xFKBP12 moieties, can be recruited to mitochondria or peroxisomes by dimerizing with FRB ^T2098L^-FIS1c or FRB ^T2098L^-PMP34 upon administration of AP21967, leading to degradation of TOIs. (ii) Ectopic expression of FIP200-binding peptide (FBP) on mitochondria by attaching it to FIS1c leads to FIP200 recruitment and mitochondrial removal. (D) Approaches following strategy IV induce proximity between TOIs and LC3B (presumably also other mATG8s) in order to tether the TOIs to phagophores, as exemplified by ATTEC. ATTEC degraders are effective on TOIs including mutant Huntingtin and other aggregates, soluble proteins like BRD4, and organelles such as LDs.

Because PROTACs are excellent examples of harnessing endogenous ubiquitination machinery to mark targets, it is natural to ask whether PROTACs can also induce autophagic degradation apart from proteasomal proteolysis. Recently, an approach recruiting IAP family E3s (IAPs, such as cIAP1, cIAP2, or XIAP) to two different OMM targets by selective non-genetic IAP-dependent protein erasers (SNIPERs), a variant of PROTAC, successfully induced mitophagy [230]; two OMM-localized proteins, HK1 and TOMM20, were fused with CRABP2, which is a protein moiety that can dimerize with IAPs in the presence of a bivalent compound, SNIPER(CRABP)-11 (Fig. 2A-i, Table 2). Administration of the compound leads to PINK1-independent mitophagy, which indicates that this system may be used in some cells where PRKN expression is low or in patients carrying PINK1 or PRKN mutations. The details, including the linkage types of ubiquitination, need to be further investigated.

**Table 2.** *Bona fide* autophagy-based degraders

Strategy	Approach	TOI	MOI	Degrader	Results	Ref
Strategy I Tagging Targets	AUTAC	METAP2	Unknown	“AUTAC1,” fumagillol attached to FBnG via a PEG linker	Targets are degraded	[32]
		FKBP12	Unknown	“AUTAC2,” SLF attached to FBnG via a PEG linker	Targets are degraded	[32]
		BRD4	Unknown	“AUTAC3,” JQ1 acid attached to FBnG via a PEG linker	Targets are degraded; degradation occurs during G_2_-to-G_1_ transition in proliferating cells	[32]
		Mitochondria (TSPO)	Unknown	“AUTAC4,” 2-phenylindole derivative attached to FBnG via a PEG linker	Targets are degraded; requires mitochondrial fragmentation but does not require PINK1	[32]
Strategy II Directly Engaging Targets	TPA	BRD4	p62	“BMF-1-64,” JQ1 acid attached to EN96 via a linker	Targets are degraded	[54]
		Mutant Huntingtin (the length of the poly-Q stretch was not reported)	p62	“BMF-1-141,” thioflavin T derivative attached to EN96 via a linker	Targets are degraded	[54]
	AUTOTAC	ERβ	p62	“PHTPP-1304,” PHTPP attached to YOK-1304 via a linker	Targets are degraded	[53]
		AR	p62	“vinclozolinM2-2204,” vinclozolinM2 attached to YOK-2204 via a linker	Targets are degraded	[53]
		METAP2	p62	“Fumagillin-105,” Fumagillin attached to YTK-105 via a linker	Targets are degraded	[53]
		tau ^P301L^	p62	“PBA-1105,” PBA attached to YTK-1105 via a linker; “PBA-1105b,” PBA attached to YTK-1105 via a longer linker; “PBA-1106,” PBA attached to YOK-1106 via a linker “Anle138b-F105,” Anle138b attached to YTK-F105 via a linker	Targets are degraded p62 oligomerization is observed The length of the linker does not influence the outcome	[53]
		Huntingtin[Q103]	p62	“PBA-1106,” “Anle138b-F105”	Targets are degraded; WT Huntingtin is not degraded	[53]
		Huntingtin[Q97]-NLS	p62	“PBA-1105,” “PBA-1106,” “Anle138b-F105,”		[53]
		Huntingtin[Q97]-NES	p62	“PBA-1106”		[53]
		Desmin ^L385P^	p62	“PBA-1105,” “PBA-1105b,” “Anle138b-F105”	Targets are degraded; WT Desmin is not degraded; the length of the linker does not influence the outcome	[53]
Strategy III Initiating Autophagy at Targets	No *bona fide* degraders have been reported yet					
Strategy IV Phagophore Tethering to Targets	ATTEC	Various Huntingtin mutants with different lengths of poly-Q stretches	LC3B*	GW 5074; ispinesib; AN1; AN2	Targets are degraded; WT Huntingtin is not degraded	[55]
		ATXN3[Q74]	LC3B*	GW 5074; AN1; AN2	Targets are degraded; WT ATXN3 is not degraded	[55]
		Various protein constructs with different lengths of poly-Q stretches	LC3B*	GW 5074; AN1; AN2	Constructs with 72Q, 53Q, 46Q, and 38Q are degraded, while the construct with 25Q is not	[55]
		BRD4	LC3B*	“10f,” JQ1 acid attached to GW 5074 via a linker	Targets are degraded	[56]
		Lipid Droplets	LC3B*	“LD·ATTEC1,” Sudan IV attached to GW 5074 via a linker; “LD·ATTEC2,” Sudan III attached to GW 5074 via a linker “LD·ATTEC3,” Sudan IV attached to AN2 via a linker; “LD·ATTEC4,” Sudan III attached to AN2 via a linker	Targets are degraded	[57]

This table presents examples from reported autophagy-based targeted degradation approaches that generated *bona fide* autophagy-based degraders, according to the strategies they follow. By using *bona fide*, we refer to degraders that bind to endogenous binding sites provided by the TOI or MOI, rather than to any additional protein moieties attached to the TOI or MOI. In fusion proteins, moieties introduced for observation purposes, such as Flag-tag and fluorescent proteins, are omitted here for simplicity. For complete information please refer to the original papers. For organelles, if a surface protein is chosen to provide the binding site, it is presented in parentheses. Names of compounds given by their developers are cited in quotation marks (e.g., “AUTAC1”).

Abbreviations: AR, androgen receptor; ATXN3, ataxin 3; BRD4, bromodomain containing 4; ERβ, estrogen receptor 2; FKBP12, 12 kDa FK506-binding protein; iLID, improved light-induced dimer; METAP2, methionine aminopeptidase 2; NES, nuclear export signal; NLS, nuclear localization signal; PINK1, PTEN induced kinase 1; SspB, stringent starvation protein B; TSPO, translocator protein; WT, wild-type.

^*^Other mATG8s may be also involved.

In contrast, mitochondrial recruitment of another E3, CRBN, via PROTAC does not trigger mitophagy. In this study, HT (HaloTag, a protein moiety that can be recognized by HTL, HaloTag ligand) was fused to TOMM20, and a PROTAC consisting of thalidomide (CRBN ligand) and HTL recruits CRBN to mitochondria. K48-polyubiquitin is observed on mitochondria, but neither LC3B recruitment nor degradation of mitochondrial markers (such as COX2) occurs (Table 2) [32].

It would be interesting to test whether or how PROTACs can induce autophagic degradation of other targets. Concerning organelles, it has been known that proteasomal proteolysis of some surface proteins, such as MFNs (mitofusins) on mitochondria [245–247], can promote autophagic degradation of the whole organelle. Thus, it might be possible to induce or enhance organelle autophagy by targeting these proteins to proteasomal degradation by PROTACs.

#### AUTAC

S-guanylation was first identified as a cellular response to *Streptococci* invasion that induces marking of the bacterial surface by K63-linked ubiquitination and subsequent autophagic degradation [137]. Later, it was demonstrated to be a sufficient signal for autophagic removal of other cargoes as well [32]. Based on this, Autophagy-TArgeting Chimera (AUTAC) was developed as a novel autophagy-based targeted degradation tool. Generally, an AUTAC consists of an FBnG (*p*-fluorobenzylguanine, a derivative of S-guanyl with better pharmacological properties and reduced perturbation on endogenous cGMP-related signaling pathways) moiety attached to a target ligand, which mimics S-guanylation and triggers K63-polyubiquitination upon binding to a target. AUTAC is effective on a variety of targets, including METAP2, FKBP12, BRD4, and mitochondria (using TSPO, an OMM protein, as a target) as illustrated in Fig. 2A-ii, and several model targets (including cytosolic, nuclear, and mitochondrial-localized) carrying the HaloTag (HT) moiety as the binding site for HTL-containing AUTACs (Tables 2 and 3).

**Table 3.** Proof-of-concept experiments for autophagy-based targeted degradation

Strategy	TOI	MOI	Methods	Results	Ref
Strategy I Tagging Targets	Mitochondria (Omp25-HT)	CRBN	A HaloPROTAC, consisting of HTL attached to thalidomide via a linker, induces dimerization between HT and CRBN, thus recruiting CRBN to mitochondria	Targets are not degraded. K48-polyubiquitin is observed on mitochondria	[32]
	Mitochondria (HK1-CRABP2 or TOMM20-CRABP2)	IAP family E3s	SNIPER(CRABP)-11, consisting of ATRA attached to MV1 via a linker, induces dimerization between IAP family E3s with the CRABP2 moiety, thus recruiting IAPs to mitochondria	Targets are degraded	[230]
	Mitochondria (Omp25-HT)	Unknown	A HaloTag-based AUTAC consisting of HTL attached to FBnG via a linker recruits an unknown MOI to HT-expressing mitochondria	Only fragmented, but not intact, mitochondria are degraded, although ubiquitination can be detected on both	[32]
	HT	Unknown	A HaloTag-based AUTAC consisting of HTL attached to Cys-S-cGMP via a linker induces dimerization between HT and an unknown MOI	Targets are degraded	[32]
	HT, NLS-HT, and NES-HT	Unknown	An improved HaloTag-based AUTAC consisting of HTL attached to FBnG, rather than Cys-S-cGMP, via a linker recruits an unknown MOI to HT	All targets are degraded; NES-HT is degraded more efficiently than NLS-HT	[32]
	Mitochondria (TOMM20[TM]-FRB ^T2098L^, with an additional Lys-rich tail)	ProxE3 (FKBP12-NEDD4[HECT])	The glue molecule AP21967 induces dimerization between FRB ^T2098L^ and FKBP12, thus recruiting ProxE3 to mitochondria	Targets are not degraded; K63-ubiquitination, p62 recruitment, retrograde transport and clustering of mitochondria are observed	[249]
	Mitochondria (FRB ^T2098L^-FIS1)	PINK1 (PINK1[Δ1-110]-2xFKBP12)	The glue molecule AP21967 induced dimerization between FRB ^T2098L^ and FKBP12, thus recruiting PINK1 to mitochondria	Targets are degraded	[253]
	Peroxisomes (FRB ^T2098L^-PMP34)	PINK1 (PINK1[Δ1-110]-2xFKBP12)	The glue molecule AP21967 induces dimerization between FRB ^T2098L^ and FKBP12, thus recruiting PINK1 to peroxisomes	Targets are degraded	[253]
	Lysosomes (LAMP1-FRB ^T2098L^)	PINK1 (PINK1[Δ1-110]-2xFKBP12)	The glue molecule AP21967 induces dimerization between FRB ^T2098L^ and FKBP12, thus recruiting PINK1 to lysosomes	Targets are not degraded, although ubiquitination is observed	[253]
	Peroxisomes (CIBN-PMP34)	CHIP (CRY2-CHIP)	CIBN moiety cand undergo a conformational change upon treatment with blue light, which enables its binding to CRY2, thus recruiting CHIP to peroxisomes	Targets were degraded; Degradation bypassed HSP70 or HSC70	[103]
	Mitochondria (TOMM20 attached to 2, 4 or 6 tandem ubiquitin moieties)	N/A	Fusion proteins consisting of TOMM20 attached to 2, 4, or 6 tandem ubiquitin moieties are ectopically expressed on the OMM. Three types of tandem ubiquitin, consisting of WT, K48R, and K0 ubiquitin, respectively, were tested	Mitochondria expressing WT or K48R constructs are not degraded Mitochondria expressing K0R constructs are degraded, and short polyubiquitin chain containing 2 ubiquitin moieties are more effective than longer ones	[230]
Strategy II Directly Engaging Targets	Peroxisomes (FRB ^T2098L^-PMP34)	NDP52 (2xFKBP12-NDP52)	The glue molecule AP21967 induces dimerization between FRB ^T2098L^ and FKBP12, thus recruiting NDP52 to peroxisomes	Targets are degraded The FIR, rather than the LIR, of NDP52 is required for degradation	[112]
	Mitochondria (FRB ^T2098L^-FIS1c)	NDP52 (2xFKBP12-NDP52)	The glue molecule AP21967 induces dimerization between FRB ^T2098L^ and FKBP12, thus recruiting NDP52 to mitochondria	Targets are degraded FIR, rather than LIR, of NDP52 is required for degradation	[112]
	Mitochondria (FRB ^T2098L^-FIS1c)	OPTN (2xFKBP12-OPTN[ΔUBAN])	The glue molecule AP21967 inducsd dimerization between FRB ^T2098L^ and FKBP12, thus recruiting OPTN to mitochondria	Targets are degraded	[230]
	Mitochondria (iLID-ActA)	AMBRA1 (AMBRA1-SspB)	iLID moiety can undergo a conformational change upon treatment with blue light, which enables its binding to sspB, thus recruiting AMBRA1 to mitochondria	Targets are degraded	[254]
	Mitochondria	AMBRA1 (AMBRA1-ActA)	ActA can localize to the OMM, thus anchoring AMBRA1 to mitochondria	Targets are degraded Mitochondrial retrograde transport and phagophore colocalization are observed	[79]
	ER (Streptavidin-CD74)	p62 (p62-SBP)	SBP can dimerize with streptavidin in an inducible and reversible manner, thus recruiting p62 to ER	Targets are not degraded; LC3B recruitment is observed	[255]
	Golgi (Streptavidin-Golgin84)	p62 (p62-SBP)	SBP can dimerize with streptavidin in an inducible and reversible manner, thus recruiting p62 to Golgi	Targets are not degraded; LC3B recruitment is observed	[255]
	LDs	p62 (TIP47[PAT]-p62 ^T352A^)	PLIN3/TIP47[PAT} can localize to the LD surface, thus anchoring p62 to LDs	Targets are degraded	[256]
Strategy III Initiating Autophagy at Targets	Mitochondria (FRB ^T2098L^-FIS1c)	ULK1 (2xFKBP12-ULK1)	The glue molecule AP21967 induces dimerization between FRB ^T2098L^ and FKBP12, thus recruiting ULK1 to mitochondria	Targets are degraded	[112]
	Peroxisomes (FRB ^T2098L^-PMP34)	ULK1 (2xFKBP12-ULK1)	The glue molecule AP21967 induces dimerization between FRB ^T2098L^ and FKBP12, thus recruiting ULK1 to peroxisomes	Targets are degraded	[112]
	Mitochondria (FRB ^T2098L^-FIS1c)	TBK1 (2xFKBP12-TBK1)	The glue molecule AP21967 induces dimerization between FRB ^T2098L^ and FKBP12, thus recruiting TBK1 to mitochondria	Targets are degraded	[112]
	Peroxisomes (FRB ^T2098L^-PMP34)	TBK1 (2xFKBP12-TBK1)	The glue molecule AP21967 induces dimerization between FRB ^T2098L^ and FKBP12, thus recruiting TBK1 to peroxisomes	Targets are degraded	[112]
	Mitochondria (FBP- FIS1c)	FIP200	FBP-FIS1c recruits FIP200 to mitochondria	Targets are degraded	[112]
Strategy IV Phagophore Tethering to Targets	Mitochondria (CISD1[MTS]-p62^[321–342])^	mATG8s	CISD1[MTS] localizes to outer mitochondrial membrane, thus anchoring p62 ^[321–342]^ to mitochondria where they recruit mATG8s	Targets are degraded	[257]
	Peroxisomes (PEX13[PTS]-eLIR)	mATG8s	PEX13[PTS] localizes to peroxisomes, thus anchoring eLIR to peroxisomes where they tether peroxisomes to phagophores	Targets are degraded	[258]
	ER (Streptavidin-CD74)	LC3B (SBP-LC3B)	SBP can dimerize with streptavidin in an inducible and reversible manner, thus recruiting LC3B to ER	Targets are not degraded	[255]
	Golgi (Streptavidin-Golgin84)	LC3B (SBP-LC3B)	SBP can dimerize with streptavidin in an inducible and reversible manner, thus recruiting LC3B to Golgi	Targets are not degraded;	[255]
	LDs (LDTS-eLIR)	mATG8s	LDTS localizes to LD surface, thus anchoring eLIR to LDs where they tethered LDs to phagophore	Targets are degraded	[258]

This table summarizes proof-of-concept experiments on autophagy-based targeted degradation according to the strategies they follow. In protein constructs, moieties that are unrelated to degradation purposes, such as Flag-tag and fluorescent proteins, are omitted here for simplicity. For full constructs please refer to the original papers. For organelles, if a surface protein is chosen to provide the binding site, it is presented in parentheses.

Abbreviations: AR, androgen receptor; ATXN3, ataxin 3; ATRA, all-*trans* retinoic acid; BRD4, bromodomain containing 4; CD74, CD74 molecule; CIBN, N terminus of Arabidopsis CIB1 (cryptochrome-interacting basic-helix-loop-helix 1); CRABP2, cellular retinoic acid binding protein 2; CRBN, cereblon; CRY2, cryptochrome circadian regulator 2; ERβ, estrogen receptor beta; FBP, FIP200-binding peptide; FIS1, fission, mitochondrial 1; FIS1c, C terminus of FIS1; FKBP12, 12 kDa FK506-binding protein; FRB, FKBP12-rapamycin binding domain of mTOR; HECT, homologous to the E6-AP C terminus; HK1, hexose kinase 1; HSP70, heat shock protein of 70kDa; HSC70, heat shock cognate 71 kDa protein; HT, HaloTag; HTL, HaloTag ligand; IAP, inhibitor of apoptosis proteins; iLID, improved light-induced dimer; LAMP1, lysosomal associated membrane protein 1; METAP2, methionine aminopeptidase 2; NEDD4, NEDD4 E3 ubiquitin protein ligase; NES, nuclear export signal; NLS, nuclear localization signal; Omp25, 25 kDa outer membrane protein; PAT, Perilipin, ADRP, and TIP47; PINK1, PTEN induced kinase 1; TIP47, perilipin 3; SBP, streptavidin-binding protein; MTS, mitochondria-targeting sequence; PTS, peroxisome-targeting sequence; PMP34, solute carrier family 25 member 17; SspB, stringent starvation protein B; TOMM20, translocase of outer mitochondrial membrane 20; TSPO, translocator protein; WT, wild-type.

Among these targets, BRD4 and a nucleus-localized model target are degraded by AUTACs despite the fact that autophagy occurs in the cytoplasm [32]. This could be explained by nuclear components entering the cytoplasm during mitosis, as degradation of BRD4 mostly occurs during the G_2_-to-G_1_ transition [32]. Another interesting observation was that the morphology of mitochondria influences the outcome of AUTACs, as only fragmented, but not intact, mitochondria can be degraded by AUTACs that target OMM proteins even though mitochondrial ubiquitination is observed in both cases, which may offer a means to distinguish between damaged and healthy mitochondria [32, 40, 248]. Mitochondria-targeting AUTACs can degrade CCCP-damaged mitochondria while sparing newly synthesized mitochondria, which can protect cells from CCCP toxicity and can also partially restore mitochondria homeostasis in fibroblasts derived from patients with Down syndrome—a genetic disease with prominent mitochondrial dysfunction [32].

Despite these exciting results, many mechanistic questions concerning AUTACs remain not well-investigated, the most critical one being how ubiquitination is induced by S-guanyl or its chemical analog; it is possible that they may directly recruit certain E3 (or E3s), in which scenario AUTACs would form ternary complexes in a PROTAC-like fashion. However, other possibilities exist and should be further tested. It is also unknown whether K63-polyubiquitination of TOIs is sufficient per se for autophagic clearance, and whether other downstream events of S-guanylation or its mimicry may also contribute to TOI degradation by autophagy.

Concerning mitochondria, it would be interesting to find out which proteins are ubiquitinated by the degraders by proteomic approaches. Another interesting finding was that mitochondrial accumulation of K63-polyubiquitin requires a considerable incubation time. In cells expressing an OMM protein fused with HT, mitochondrial accumulation of K63-polyubiquitin does not occur until 8 h after incubation with the FBnG-HTL degrader. At 8 h, mitochondrial K63-polyubiquitin dramatically increases, and remains at the level afterwards. A similar time-course was also recorded using another degrader, AUTAC4, which binds to the OMM protein TSPO. No mitophagy was observed in both studies. Due to the rapidity of enzymatic reactions, it is likely that ubiquitination of OMM proteins does occur prior to the time point when mitochondrial accumulation of ubiquitin is detected, but they are somehow removed. The underlying mechanisms could be studied in the future.

Other questions include whether AUTACs influence autophagy flux, whether the degraders induce off-target degradation as measured by proteomics, whether changing the linker would affect the outcomes, and whether this technology could be applied to a broader spectrum of TOIs, including protein aggregates and intracellular pathogens. A hook effect, which is typical for bivalent degraders where elevated levels of the linker interfere with ternary complex formation [11], was not obvious; it is uncertain whether this may imply a different mode of action or be simply due to the limited range of concentration tested, and further investigations are needed. While AUTACs binding to TSPO removed the whole mitochondria, whether and how membrane proteins can be degraded individually would be interesting to investigate. Moreover, it is necessary to confirm the effects of AUTACs *in vivo*.

#### Other experimental approaches

Apart from PROTACs and AUTACs, there are other reported approaches that induce autophagic degradation of various targets by ubiquitination. For instance, artificially tethering STUB1/CHIP, an E3, to peroxisomes using a light-induced dimerization CRY2-CIBN system also yields degradative effects [103]. CRY2, which was attached to CHIP in this study, can undergo a conformational change upon blue light treatment and bind to peroxisome-localized CIBN. Blue light-induced recruitment of CHIP triggers peroxisomal ubiquitination, p62 recruitment, and autophagic degradation (Fig. 2A-iii, Table 2) [103].

Another individual approach utilizes a modified E3 ligase, proximity-induced E3 (ProxE3). This E3 was constructed by fusing a HECT domain from NEDD4 (an E3), specifically catalyzing K63-polyubiquitination, with an FKBP12 moiety [249]. As a well-established chemically induced dimerization (CID) system [250], an FKBP12 moiety can dimerize with an FRB ^T2098L^ moiety in the presence of a glue molecule (AP21967), which is used to recruit ProxE3 to desired targets [251]. In this case, FRB ^T2098L^ is fused with an OMM protein and carries an additional lysine-rich tail for more efficient ubiquitination (Table 2) [249]. Upon AP21967 treatment, recruitment of ProxE3 to mitochondria leads to mitochondrial K63-polyubiquitination, p62 recruitment, and perinuclear clustering of mitochondria; however, mitochondrial degradation is not observed [249]. Thus, it seems that although ProxE3-induced K63-ubiquitination may be sufficient for p62 recruitment and mitochondrial clustering, but not for mitophagy, consistent with an earlier observation [252]. The effects of mitochondrial fragmentation could be tested here in the future.

In a less direct route, one study tested the outcomes of artificially recruiting PINK1, a kinase responsible for sensing mitochondrial depolarization and recruiting Parkin in the PINK1-Parkin pathway for mitophagy [82,102,131–133], to targets including mitochondria, peroxisomes, and lysosomes by the FKBP12-FRB ^T2098L^ CID system. In this study, two FKBP12 moieties are fused to a PINK1 that lacks its N-terminal 110 residues responsible for mitochondrial localization, while FRB ^T2098L^ is attached to organelle surface markers including FIS1, PMP34, and LAMP1 [253]. AP21967 treatment leads to PINK1 localization to all three targets, followed by Parkin recruitment and ubiquitination of targets (Fig. 2A-iii, Table 2); However, only mitochondria and peroxisomes are degraded, while lysosomes are not [105, 253]. This renders a possibility that lysosomal ubiquitination per se is not sufficient to trigger its autophagic removal, which may require other factors.

Ectopically expressing tandem ubiquitin chains on mitochondria can also lead to mitophagy [230]. Tandem ubiquitin chains, in which 2, 4, or 6 ubiquitin moieties are attached head to tail to mimic M1-polyubiquitin, are attached to an OMM protein for mitochondrial localization. Three types of tandem ubiquitin chains were tested, consisting of wild-type (WT) ubiquitin, K48R ubiquitin, and K0 ubiquitin, respectively, in which K0 ubiquitin had all lysine residues mutated to arginine, making it inaccessible to further branching by cellular enzymes [230]. The results showed only K0 chains succeed in inducing mitophagy, which indicates that linear polyubiquitin may function as a sufficient tag for mitophagy (Fig. 2A-iii, Table 2) [230]. Shorter chains with 2 tandem ubiquitin molecules, which recruit p62, NBR1, and OPTN in this study, are more potent in inducing mitophagy than longer chains. Unlike AUTAC, mitochondrial degradation in this study does not require artificial induction of mitochondrial fragmentation. Whereas this approach has been successful, developing autophagy-based degraders from it may be difficult; even though E3s that specifically build linear polyubiquitin exist (such as LUBAC), preventing linear polyubiquitin from branching in a clinically acceptable way may be so far difficult to achieve. Nevertheless, it may be plausible to test the outcomes of recruiting such linear-specific E3s.

### Strategy II: directly engaging targets

#### TPA

Cargo receptors are responsible for recognizing cargoes with or without tags to establish cargo selectivity, which makes directly recruiting cargo receptors to the target another appealing idea to induce target degradation by autophagy. This idea has been implemented by the Nomura group, which patented a technology termed TPA in 2019 [54]. This technology involves a series of bivalent compounds that can covalently bind to cargo receptors (and alternatively, other autophagy-related proteins) by one end, while binding to the target by the other end. Two compounds, BMF-1-64 and BMF-1-141, are revealed in their patent; these compounds are constructed by attaching EN96, a covalent ligand to p62, to JQ1 acid and thioflavin T derivative (a pan-aggregate binding ligand), respectively [54]. When tested in cells, these compounds succeed in degrading BRD4 and mutant Huntingtin (Fig. 2B-i, Table 3) [54]. This patent also reveals covalent ligands for OPTN, ALFY, and others, which may be utilized for developing more autophagy-based degraders.

#### AUTOTAC

In 2022, AUTOphagy-TArgeting Chimera (AUTOTAC) was reported as a novel autophagy-based degrader that harnesses p62 [53]. AUTOTACs were inspired by N-degrons, which serve not only as binding tags recognized by p62, but also activators of p62 by enabling its oligomerization [108, 174, 259]. Based on their earlier work [98, 174], structure-activity relationship studies using the ZZ domain of p62 were first conducted to identify Nt-Arg-mimicking ligands (known as autophagy-targeting ligands, ATL, in this study). This yielded 4 ligands: YOK-2204, YOK-1304, YTK-105, and YT-8-8, which are confirmed to promote p62 oligomerization while also increasing autophagy flux, as measured by p62 and LC3B turnover rate [53]. Several other p62 ligands with structural similarities (YTK-1105, YOK-1106, and YTK-F105) are also used in this study, but how they were identified and evaluated was not mentioned [53].

The technology was first tested on ERβ, AR, and METAP2. Administration of AUTOTACs (PHTPP-1304, vinclozolinM2-2204, and Fumagillin-105, respectively) leads to colocalization between targets and phagophore markers, accompanied by robust degradation of all three targets, as shown in Fig. 2B-ii, Table 3. As expected, this effect does not require targets ubiquitination. AUTOTACs are more potent on inhibiting downstream signaling pathways of target proteins, such as cell survival and migration, than the target ligands per se; for instance, the IC_50_ for PHTPP-1304 is ~5-fold lower than for PHTPP alone. AUTOTACs also display persistent post-washout degradation, suggesting degrader recycling from lysosomes [53]. These effects are similar to what is observed in PROTAC [20–22].

Next, misfolded proteins and aggregates were chosen as targets. Two ligands, 4-phenylbutyric acid (PBA) and Anle138b, were used as target-binding ligands; the former binds to exposed hydrophobic regions, a hallmark for misfolded proteins, whereas the latter recognizes oligomeric species of misfolded proteins (summarized in Table 3) [53]. Their attachment to p62-binding ligands gives rise to four AUTOTACs: PBA-1105, PBA-1106, Anle138b-F105, and PBA-1105b. When tested on a mutant of Desmin that is known to aggregate, PBA-1105, PBA-1105b, and Anle138b-F105 proved effective while sparing the WT, nonaggregating Desmin. Other aggregate-prone mutant proteins, including tau ^P301L^, Huntingtin[Q97]-NLS, Huntingtin[Q97]-NES, and Huntingtin[Q103], can also be selectively removed by AUTOTACs, while WT tau and Huntingtin are spared (Table 3); When different concentrations of degraders are tested, a hook effect is observed. Moreover, PBA-1105 removes aggregates of mutant tau from mouse brains in a dose-dependent manner [53]. However, correlating behavioral benefits of aggregate clearance were not studied.

AUTOTACs are somewhat special as they utilize allosteric activators as p62 ligands, which may also apply to other autophagy-based degraders that harness cellular machinery that require activation, such as ULK1 or Parkin. AUTOTACs are potent on degrading aggregating or soluble nuclear targets, a possible result from nuclear-cytoplasmic shuttling of p62 or release of nuclear components during mitosis, the mechanisms of which could be further studied. While p62 ligands were confirmed to bind to p62 and induce its oligomerization, their affinity to p62 was not measured and the ternary complex of TOI-degrader-p62 was not directly observed, which could be evaluated in the future. The study also concluded that linker length does not significantly influence degradative effects by comparing two degraders, PBA-1105 and PBA-1105b, with identical ligands but attached by linkers with different lengths (Fig. 2B-ii). Importantly, TOI degradation does not require the UBA domain of p62 responsible for ubiquitin binding, which indicates that AUTOTAC may be applicable to pathologies where the ubiquitin-binding function of p62 is disturbed, as observed in some cases of Paget disease of bone [260] and amyotrophic lateral sclerosis/ALS [261].

As AUTOTACs increase p62 turnover and autophagy level, it is possible that degradation of non-TOI cargoes, such as KEAP1 and other ubiquitinated proteins, may be also enhanced. This effect may be beneficial in cases where overall activation of autophagy is welcomed as in some neurodegenerative diseases [262, 263], or where KEAP1 inhibition is desirable [264], but may also introduce unintended off-target effects in other circumstances. Meanwhile, AUTOTACs may also compete p62 from its natural cargoes that are recognized via Nt-Arg [108], hindering their degradation. To test these possibilities, it is plausible to evaluate the effects of AUTOTACs on a proteomic level. So far, AUTOTACs have not been reported to degrade organelles, nor have the degraders been verified in animal models, which could be focused on by future studies.

#### Other experimental approaches

Much attention has been paid to p62, the prototypic cargo receptor. In 2018, Itakura *et al*.constructed a chimeric protein consisting of a TIP47-derived PAT domain, which bind to LDs, and p62 ^T352A^. The point mutation was introduced to abolish the p62-KEAP1 interaction for avoiding unintended alteration of the KEAP1-NRF2 pathway (Fig. 2B-iii, Table 2) [256]. The construct localizes to LDs and induces autophagy-dependent lipolysis, indicated by reduction of LD size and quantity, accompanied by a decreased triglyceride level [256].

While recruiting p62 to some targets yields degradative effects, it does not seem sufficient to target the ER or Golgi to autophagic degradation as observed in a recent study [255]. To control recruitment of p62 to targets, this study utilized a dimerization system in which streptavidin can dimerize with streptavidin-binding protein (SBP) in an inducible and reversible manner. SBP-fused p62 can be localized to the ER or Golgi by binding to streptavidin-fused organelle markers, CD74 and Golgin84, respectively [255]. Localization of p62 is followed by recruitment of LC3B to the organelles. However, neither autophagosome formation nor recruitment of lysosomes is observed for either target. Degradation of ER or Golgi proteins is also absent (Table 2) [255].

Apart from p62, other cargo receptors have also been tested. Recruiting NDP52 to mitochondria or peroxisomes via the FKBP12-FRB ^T2098L^ CID system successfully targets these organelles to autophagic degradation [112]. Of note, this degradative effect is observed in cells lacking PINK1 or mATG8s; furthermore, a NDP52 construct that is depleted of its SKICH domain, which is responsible for recruiting TBK1 and FIP200, fails to induce mitophagy even though it still bound to mATG8s (Table 2).

Recruiting OPTN to mitochondria using the same CID system also induces mitophagy [230]. OPTN used in this experiment was deprived of its ubiquitin-binding domain to avoid unintended ubiquitin-dependent recruitment. In this case, mitochondrial recruitment of OPTN is followed by colocalization between mitochondria and ATG9A, which is required for OPTN-induced mitophagy (Fig. 2B-iii, Table 2) [230].

Autophagy and Beclin-1 regulator 1 (AMBRA1) was reported to be a cargo receptor recruited to mitochondria in a Parkin-dependent mechanism [265]. Ectopically expressing AMBRA1 on mitochondria by fusing AMBRA1 to ActA (actin assembly inducing protein from *L.monocytogenes*), an OMM-localizing peptide, induces mitophagy in a Parkin- and p62-independent manner [79]. Later, the same group developed an optogenetic system that allows for reversible, light-controlled recruitment of AMBRA1 to mitochondria, which is also successful for inducing mitophagy (Fig. 2B-iii, Table 2) [254].

### Strategy III: initiating autophagy at targets

In 2019, a series of approaches that artificially tether upstream autophagy machinery and regulators to mitochondria or peroxisomes using CID-based protein constructs were reported by the Youle team [112]. It was shown that artificial recruiting of ULK1 to mitochondria or peroxisomes is sufficient to trigger autophagic clearance of these organelles without requiring global inhibition of mTOR or activation of AMPK, which are important upstream pathways regulating ULK1. The effect was suggested to result from ULK1 autophosphorylation and activation upon recruitment and clustering on organelle surfaces (Fig. 2C, Table 2) [112].

The Youle team also demonstrated that recruitment of FIP200, another component of the ULK1 complex, makes it possible to induce ULK1 complex assembly at the site of the target and subsequent target degradation. They constructed a fusion protein containing an ATG16L1-derived FIP200-binding peptide (FBP; 100-250 amino acids of ATG16L1) and FIS1c, which localizes to mitochondria and induces mitophagy [112]. When this peptide is attached to E3-dead PRKN, which can still translocate to mitochondria in response to mitophagy stimuli but cannot catalyze ubiquitination, it can mediate mitophagy by recruiting the ULK1 complex to mitochondria. This effect is observed even in cells lacking NDP52 or TBK1, which are normally required for recruiting ULK1 in mitophagy (Fig. 2C, Table 2) [112]. Artificially localizing TBK1 to mitochondria and peroxisomes via CID also yields degradative effects in an ULK1-dependent manner (Fig. 2C, Table 2) [112].

### Strategy IV: tethering phagophores to targets

#### ATTEC

The function of tethering cargos to phagophores can be mimicked by bivalent degraders that simultaneously bind targets and mATG8s (or other docking sites on the phagophore). In 2019, Lu *et al*. reported two glue molecules that simultaneously bind to mutant Huntingtin and LC3B, screened from a library of >3000 compounds [55]. The compounds, GW 5074 and ispinesib, can selectively bind to mutated Huntingtin with an extended poly-Q stretch, but not WT Huntingtin. Consistent with *in vitro* results, these compounds lower mutant Huntingtin levels but spare WT Huntingtin when applied to primary cortical neurons derived from a murine model of HD without altering the cellular autophagy level. As expected for bivalent degraders, a hook effect was observed. An examination of chemicals with structural similarities identified two additional hits, AN1 and AN2, with similar effects (Fig. 2D).

This type of degrader, termed AuTophagosome TEthering Compounds (ATTEC), was also tested on several artificial targets carrying poly-Q stretches with different lengths, and the studies concluded that a recognition threshold lies somewhere between 25Q and 38Q (see Table 3) [30, 55]. ATTECs are protective against toxicity induced by mutant Huntingtin in iPSCs derived from HD patients. When tested in animal models, ATTECs extend the lifespans of flies expressing mutated Huntingtin, and improve behavioral outcomes in a murine HD knock-in model. ATTECs are also effective against other expanded poly-Q-containing targets, such as mutant ATXN3 [55].

More recently, the same team succeeded in targeting LDs to autophagic degradation using LD-targeting ATTECs. These ATTECs are modular, and are constructed by attaching Sudan III or Sudan IV (lipid stains) to GW 5074 or AN2, the LC3B-binding compounds reported in their previous work, via a linker (Fig. 2D) [57]. A reduction of LD numbers is observed accompanied by colocalization of LDs and lysosomal markers, which indicates lipolysis. When the compounds were tested on mouse embryonic fibroblast cells, increased β-oxidation is observed, while the free fatty acid level in the culture medium is not significantly altered [57]. Moreover, ATTECs significantly reduce the body weight, number, and size of LDs in livers, as well as triacylglycerol and total cholesterol levels in livers and sera, when applied to obese mice and NASH mice [57]. Interestingly, this study also examined formation of a triacylglycerol-ATTEC-LC3B ternary complex via a modified ELISA assay. Reconstituted lipid droplets (adiposomes) may be another choice for assessing ternary complex, as they are structurally more similar to cellular LDs [266]. This could be tested in future studies.

Another team also succeeded in targeting BRD4 to autophagic degradation using a degrader consisting of a BRD4 ligand and GW5074 (Fig. 2D) [56]. Treatment with this compound, 10f, induces a robust decrease in BRD4 level in a dose-dependent manner, which becomes apparent as early as 2 h after administration. However, no hook effect was reported in the given range of concentration. Colocalization of BRD4 with LC3B in cytoplasmic puncta may suggest a portion of BRD4 becomes somehow “trapped” in the cytoplasm [56]. How this occurs is not well understood and should be further evaluated. It is known that BRD4 can be released during mitosis, as observed in AUTAC [32]. Meanwhile, a portion of mATG8s is reported to shuttle from the nucleus to the cytoplasm, which is involved in delivering some of its nuclear binding partners, such as SIRT1 and Lamin B1, to autophagic degradation [267–269]. Thus, a hypothesis is that BRD4 degradation in this study may follow a similar mechanism.

While degradation is observed for several targets, there are still some questions unsolved for ATTECs. To date, all reported ATTECs do not alter autophagy flux [55–57]; however, they may compete with the endogenous binding partners of mATG8s, most importantly cargo receptors, and thus an influence on the degradation of other autophagic cargoes is of particular concern. Meanwhile, it has been reported that mATG8s have nonautophagic functions, such as coating EDEMosomes [270] and loading cargoes into extracellular vesicles [271]. Whether these activities may also be involved in lowering of cellular TOI levels could be further studied. While ATTECs are capable of tethering TOIs to lipidated mATG8s that coat the phagophore, they also interact with free, unlipidated mATG8s. Therefore, it is possible that ATTECs first recruit soluble mATG8s to the cargo, before these mATG8s are attached to an expanding phagophore. This effect may be more prominent in cells with a lower basal autophagy level, or in cases involving nuclear TOIs, where it might be beneficial for TOI degradation.

It would be also important to elucidate how the currently used LC3B ligands bind to LC3B, presumably also to other mATG8s [55], and whether other reported ligands of mATG8s may also function well in ATTEC degraders [238, 239]. Whether ATTECs are catalytic is another question yet to be confirmed. In a kinetic model for degradation of mutant Huntingtin by ATTECs, degraders are presumed to be recyclable from lysosomes, but this has not been experimentally confirmed by, for instance, observing post-washout degradation [272]. Degradation of other TOIs, such as LDs and BRD4, likely follow different kinetics, which can be evaluated as well.

#### Other experimental approaches

In 2022, a team designed a p62-based construct to induce mitophagy to reduce mitochondria carryover during mitochondria replacement therapy, a technique that transfer nuclear materials from the donor zygote to a recipient zygote to remove defective donor mitochondria [257,273–275]. The construct contains two major functional modules: a mitochondria-targeting sequence (MTS) from CISD1 (CDGSH Iron Sulfur Domain 1) and a p62 segment that covers its LIR (p62_321-342_). When expressed, mitochondria were found to aggregate at perinuclear regions in the early phase of transfection, before robustly degraded [257]. The mitophagy-inducing effect was not accompanied by significant alterations on mitochondrial membrane potential, cellular ROS levels, or mitochondrial proliferation. The offspring generated from the mouse embryos also exhibited little changes in growth, reproduction, and behavior compared with WT mice [257].

In a recent preprint, an attempt at localizing an mATG8-interacting segment from the cargo receptor OPTN to LDs succeeded in targeting LDs to autophagic degradation [258]. The protein construct basically contains OPTN[120-190], a segment proven sufficient for interacting with mATG8s, attached to a PLIN1-derived LD-targeting sequence (LDTS). Some mutations were introduced to enhance the mATG8-binding ability of this segment, including phospho-mimetic substitutions upstream of its LIR, and a point mutation at the first residue in the LIR from Phe to Trp (see Table 2). The modified OPTN segment is named engineered LIR (eLIR) [258]. The construct, LDTS-eLIR, localizes specifically to LDs and induces lipophagy in cultured cells. When the construct is specifically expressed in mouse liver using an adeno-associated virus vector, it lowers liver weight and markedly alleviates NASH pathology including liver steatosis and fibrosis, while leaving the serum lipid profile mostly unaltered [258]. In addition, attaching eLIR to a peroxisome-targeting sequence from PEX13, which targets the construct to peroxisomes, also induces pexophagy [258].

The team that recruited p62 to ER and Golgi via the SBP-streptavidin inducible dimerization system also tried recruiting LC3B to these targets, but no autophagic degradation is observed (Table 2) [255]. A possible explanation could be that recruited LC3B constructs are neither sufficient to activate the whole autophagosome biogenesis program, nor sufficient to induce phagophore engulfment of target compartments [255]. Because reticulophagy is usually accompanied by ER fragmentation [83, 182], it would be interesting to test whether inducing ER fragmentation additionally would promote its degradation by this approach.

## Future perspectives

**Figure 3.**
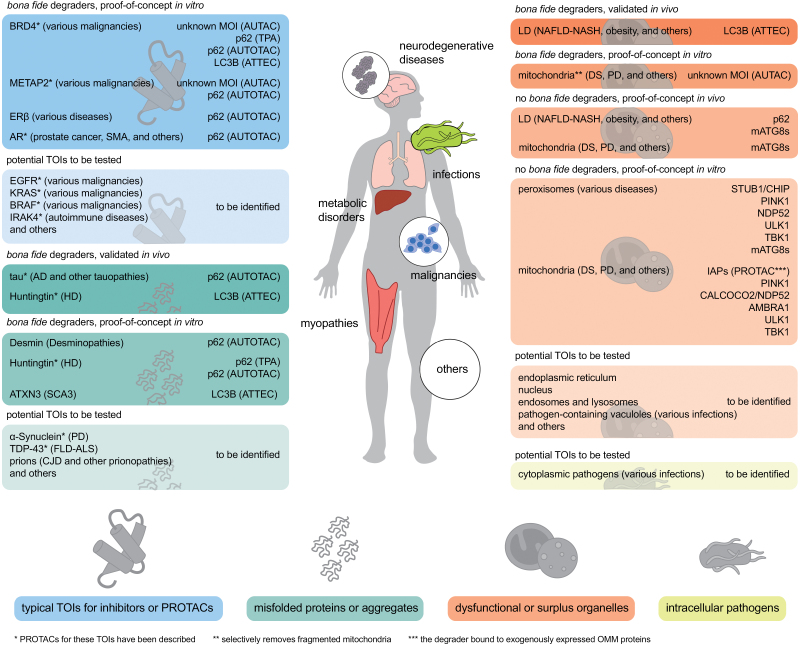
Potential implications of autophagy-based degradation in diseases. This figure presents TOIs that have been successfully degraded by autophagy-based targeted degradation approaches, as summarized in Tables 2 and 3, their current progress of development, and their implications in human diseases. Abbreviations: AD, Alzheimer’s disease; AMBRA1, autophagy and Beclin-1 regulator 1; AR, androgen receptor; ATXN3, ataxin 3; BRD4, bromodomain containing 4; NDP52, calcium binding and coiled-coil domain 2; CJD, Creutzfeldt-Jakob disease; DS, Down syndrome; EGFR, epidermal growth factor receptor; ERβ, estrogen receptor beta; FLD-ALS, frontotemporal degeneration-amyotrophic lateral sclerosis; HD, Huntington’s disease; IAPs, inhibitor of apoptosis proteins; IRAK4, interleukin 1 receptor-associated kinase 4; LD, lipid droplet; LC3B, microtubule associated protein 1 light chain 3 beta; mATG8s, mammalian Atg8-family proteins; METAP2, methionine aminopeptidase 2; MOI, machinery-of-interest; NAFLD, nonalcoholic fatty liver disease; NASH, nonalcoholic steatohepatitis; OMM, outer mitochondrial membrane PD, Parkinson’s disease; PINK1, PTEN induced kinase 1; SCA3, spinocerebellar ataxia type 3; SMA, spinobulbar muscle atrophy; p62, sequestosome 1; CHIP, Carboxy terminus of Hsp70-interacting protein; TDP43, TAR DNA binding protein of 43kDa; TBK1, TANK binding kinase 1; TOI, target-of-interest; ULK1, unc-51 like autophagy activating kinase 1.

**Figure 4.**
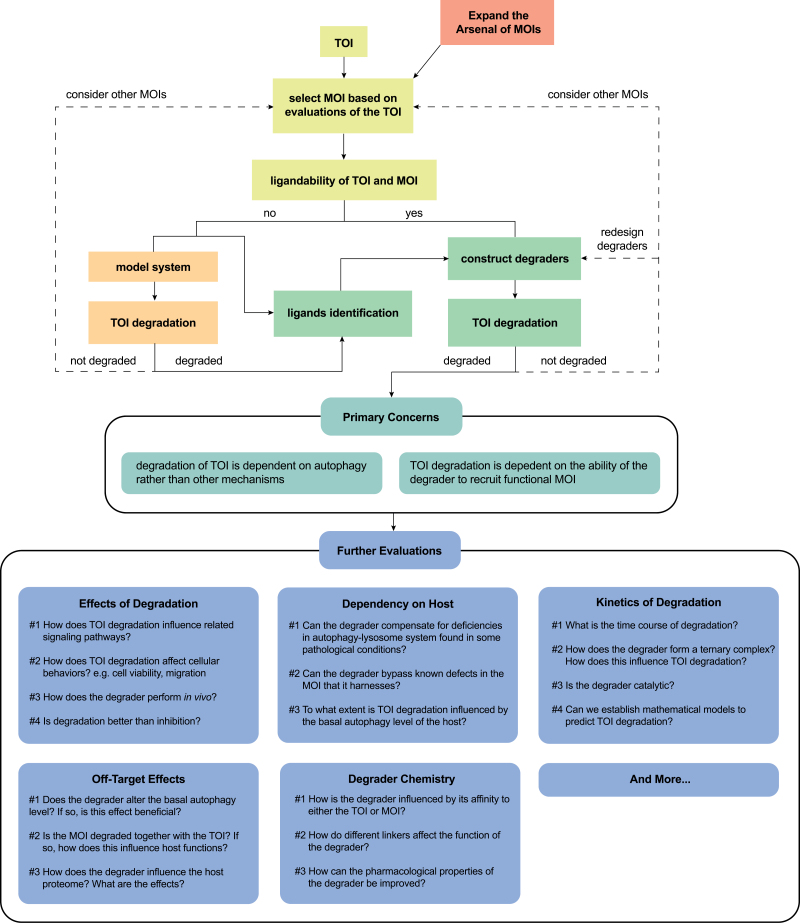
Developing and evaluating autophagy-based degraders. The workflow for generating an autophagy-based degrader starts from evaluating the properties of the TOI and selecting MOIs from a cellular arsenal (yellow panels and the red panel). If both the TOI and MOI are ligandable, the ligands could be attached by a linker to convert them into a bivalent compound (green panels). If no ligands are applicable for the TOI or MOI, the compatibility of the pair could be primarily evaluated in a model system (orange panels). Validated pairs then undergo ligand identification in order to obtain a bivalent compound. Once obtaining the bivalent compound, cellular validation can be applied to the compound to test whether it can induce autophagic degradation of the TOI in a process requiring recruitment of a functional MOI (cyan panels). If these tests are not passed, the bivalent compound can be redesigned, or another MOI can be selected to repeat the development process. Validated bivalent degraders can be then subjected to further evaluations based on their other properties.

Autophagy-based targeted degradation tools, such as AUTAC, TPA, AUTOTAC, and ATTEC, are emerging rapidly and have greatly expanded the landscape of targeted degradation. Together with other lysosome-based targeted degradation tools, such as LYTAC, MODE-A, and AbTAC, these promising modalities not only offer an alternative route to deal with the druggable or PROTACable targets, but also provides a promising choice to degrade many novel targets, especially dysfunctional organelles or cytoplasm-invading pathogens, or targets that may be less efficient to be countered by other means, such as protein aggregates, as summarized in Fig. 3. Apart from them, a number of proof-of-concept designs have been proven effective on various targets as well. Taken together, these approaches have paved paths towards clinical applications of autophagy-based degraders. In this section, we briefly introduce a workflow for development and clinical translations of novel autophagy-based degraders as seen in Fig.4, while also presenting some important issues that may emerge during the process.

The cell employs a considerable variety of machinery, including tagging machinery, cargo receptors, scaffold proteins, autophagy machinery, and others, to mediate selective autophagy (Table 1). All of them are possible candidate MOIs that may be utilized for targeted degradation purposes, which endows autophagy-based targeted degradation tools with significantly more possible modes of action than E3-based PROTACs. However, only a small fraction of candidate MOIs have been tested to date (Table 2, Fig. 3). Thus, we suggest that future studies could expand the arsenal of MOIs by including other yet-untested MOIs into evaluations (Fig. 4, red panel).

### Choosing MOIs based on evaluations of TOIs

The workflow for generating autophagy-based degraders starts from evaluating the TOI. Whereas TOIs for proteasomes are limited to proteins, TOIs for autophagic degradation fall in a significantly wider range, including soluble proteins, aggregates, organelles, and cytosolic pathogens, which vary in their chemical nature, size, structure, subcellular localization, biological behavior, and many other properties that may affect degradation (Fig. 4). An example has been provided by AUTAC: when a model target is localized to the cytoplasm and nucleus, by adding an NES or NLS, respectively, the cytoplasmic target is degraded more efficiently than the nucleus-localized target [32]. Still, whether and how properties of TOIs influence autophagic degradation have not been well understood in general and deserve greater attention.

The properties of TOIs may also influence their compatibility with MOIs. Because so many MOIs with different functions exist, a natural hypothesis is that, for a given TOI, the effects of recruiting different MOIs would vary, and an optimal MOI with the highest degradation efficiency would exist. It would be of significance to systematically compare different combinations of TOIs and MOIs, perhaps in a model system, to find out how TOI properties would influence their preference to MOIs. This may guide MOI choice for a given type of TOI in the future (Fig. 4, yellow panels).

When organelles are chosen as TOIs, a surface protein is usually chosen to provide a binding site, which enables degradation in some reported cases (Tables 2 and 3). The same principle may apply to protein complexes as well, in which targeting a subunit may remove the whole complex. However, whether or how a membrane protein or a subunit can be individually targeted without affecting the whole organelle is currently unknown. It is also known that some pathogens can evade destruction by interfering with various steps in autophagy, and whether they could be targeted via autophagy is uncertain [38]. In addition, some aggregates could be lysosome resistant and may even propagate between cells via exocytosis of lysosomes. Applying autophagy-based degradation tools on such TOIs should be accompanied with caution.

### Developing and assessing bivalent compounds

Autophagy-based degraders and PROTACs share the common nature of bivalent compounds [40], which come in two forms: modular degraders, which are constructed by attaching two monovalent ligands via a linker [276]; and nonmodular degraders, known as molecular glues, which are bivalent by themselves and require no linker (see Fig. 2 legend) [277, 278]. While modular degraders are constructed based on a knowledge of the individual ligands and thus can be designed rationally, many molecular glues were identified by screening, as were the mutant Huntingtin-targeting ATTEC compounds [55, 277–280]. In either way a bivalent compound capable of binding to both the TOI and the MOI is obtained (Fig. 4, green panels). Methodologies and technologies for identifying ligands, evaluating linkers, and constructing such compounds have been well-developed and discussed extensively elsewhere [1, 8, 13, 41, 281–286].

The compound could be first applied to test systems such as cultured cells to assess whether it can induce TOI degradation, measured primarily by a decrease in protein level (Fig. 4, green panels). If a compound does not induce any decrease in the protein level, as measured by immunoblotting or other means, it is unlikely to function and could be redesigned, by changing the attachment points for linkers on the ligands, changing linkers in structure, or switching to other ligands. Otherwise, it may indicate that the currently tested MOI may not be compatible with the TOI at all. Multiple rounds of designing may be required before a compound is validated (Fig. 4, rightward dashed arrows) [276]. During this process, it is important to set up controls carefully to rule out other causes of lowered protein level, such as reduced gene expression, and to verify that degradation occurs via the autophagy-lysosome pathway rather than other routes, such as proteasomes (Fig. 4, cyan panels). For modular compounds, the degradation should be also dependent on the bivalent compound rather than any of the individual ligands to rule out ligand-induced instability and degradation of the TOI. Degradation should also be dependent on a functional MOI that is recruited by the compound.

### Optional: evaluating TOI-MOI pairs in model systems

Only a few approaches have generated *bona fide* degraders to date, while the rest and majority utilize model systems of CID, LID, or ectopic expression to induce TOI proximity with MOIs. Due to the fact that the concentration of the dimerization agent in a CID system can be titrated, CID models, especially knock-in models that express the TOI at the endogenous level, are preferred. Such model systems could be applied to rule out some candidate MOIs that are unlikely to induce TOI degradation or used in scenarios where either the TOI or MOI lacks proper ligands (Fig. 4, orange panels and leftward dashed lines). In canonical CID systems like FKBP12-FRB ^T2098L^, neither the TOI nor MOI require ligands; otherwise, systems similar to dTAG or HaloPROTAC could be used, where only one protein needs to be ligandable while the other one is fused to an FKBP12 ^F36V^ moiety or a HT moiety, binding to AP1867 or HTL, respectively [287–289]. Alternatively, LID systems may be used to mark a portion of the cytoplasm to see whether the TOIs in this region can be selectively removed, while TOIs in the unilluminated regions are unaffected. For MOIs validated in model systems, such as CHIP, NDP52, and ULK1, identifying their ligands and generating degraders would be the following steps (Fig. 4, green panels).

### Further evaluations and special concerns

Some properties exhibited by PROTAC, such as the hook effect, catalytic degradation, and advantages of degradation over inhibition by the target ligand per se [2], are also expected for autophagy-based degraders (Fig. 4, blue panels). Indeed, some of these traits have been observed in some degraders and should be further evaluated in others to better understand their pharmacological behaviors [53, 55, 57]. Meanwhile, autophagy-based degraders are also expected to encounter unique issues, as briefly discussed in this section.

Basically, the effectiveness of degraders would be dependent on functionality of the host autophagy-lysosome pathway, which can be compromised in disease conditions including lysosomal storage disorders, neurodegenerative diseases, and others [43, 290–292]. It is important to assess whether degraders can compensate for the defects in these scenarios, for instance, by bypassing the affected machinery or restoring its functions. It is also possible to combine targeted degradation with other therapeutic approaches, such as autophagy modulators or enzyme replacement therapy [43].

While a single E3 molecule is theoretically sufficient to ubiquitinate a TOI molecule in PROTAC, multiple copies of some MOIs may be required to crowd at the TOI to efficiently trigger autophagic degradation. For example, it was speculated that a cluster with at least 30 ULK1 molecules is necessary to form early autophagic structures [293], which may pose a quantitative threshold for ULK1-harnessing degraders. Recruitment of ULK1 by CID is sufficient to trigger autophagic removal of mitochondria or peroxisomes [112]. Because these TOIs provide multiple binding sites for multiple ULK1 molecules, it is possible that recruited ULK1 may spontaneously cluster and become activated, and it would be interesting to test whether ULK1 can also effectively target TOIs that do not encourage spontaneous crowding. Here, our hypothesis is that MOIs with quantitative thresholds may require a minimum “density” of binding sites of the TOI to be efficiently activated. In addition, the stoichiometry of the “ternary complex” in this scenario is unlikely to be 1:1:1, as in PROTACs [294]. Assessing such MOIs and their ternary complexes *in vitro* may require novel methods.

PROTAC is featured by event-driven pharmacology, in which transient formation of ternary complexes, rather than prolonged occupancy of TOI active sites by inhibitors, is sufficient to induce TOI ubiquitination and degradation [6, 10]. This may also apply to autophagy-based degraders that induce enzymatic modifications of TOIs. However, this may not be the case for other types of autophagy-based degraders. For instance, it is likely that ATTECs shall interact with both TOI and mATG8s continuously to maintain a proximity between them, though they also do not have to occupy the active site because the silencing effect is provided by degradation, rather than inhibitory binding. Further studies are needed to verify this hypothesis.

LLPS is involved in multiple aspects of autophagy, such as regulation of the autophagy machinery and coaggregation of cargoes with cargo receptors [153, 154, 240, 242]. It would be important to evaluate whether LLPS is also implicated in degradation induced by autophagy-based degraders, by assessing whether they would incorporate TOIs into phase-separated condensates, and whether they could also remove aggregates or inclusions with low fluidity seen in some neurodegenerative diseases [295–297].

## Conclusion

To date, targeted degradation tools like PROTACs have successfully harnessed the ubiquitination-proteasome system. To expand the field of targeted degradation, novel methodologies that manipulate the autophagy-lysosome system have emerged, showing great potential to degrade a significantly wider range of targets, including aggregating proteins, dysfunctional organelles, cytoplasmic pathogens, and others. However, the potential of this pathway for targeted degradation has been far less than fully excavated. Understanding the mechanisms of selective autophagy, the pathways that target cellular cargoes for autophagic degradation, can inspire the design and development of novel bifunctional degraders that trigger target degradation by autophagy.

Delivery of cellular cargoes to selective autophagy requires two steps: recognition of cargo, which distinguishes the cargo from other cellular materials; and establishment of cargo-phagophore proximity, which ensures cargo engulfment by the phagophore. These two steps form the basis of autophagy-based degraders, from which various strategies and approaches have emerged. We have proposed four major strategies for autophagy-based degraders: *Tagging Target*, *Directly Engaging Target*, *Initiating Autophagy at Target*, and *Phagophore-tethering of Target*, which correspond to the major mechanisms involved in establishing cargo selectivity. While the process towards novel autophagy-based degraders will be inspired and aided by methodologies utilized for developing PROTAC and other degraders, unique and novel problems are expected to emerge. Therefore, we also provide a workflow that may guide development and validation of autophagy-based targeted degradation tools.

In summary, we have presented a mechanism-based blueprint that may inspire the development of novel autophagy-based degraders, which would be the next major step in the field of targeted degradation.

## References

[1] Paiva SL, Crews CM. Targeted protein degradation: elements of PROTAC design. Curr Opin Chem Biol 2019;50:111–19. 31004963 10.1016/j.cbpa.2019.02.022 PMC6930012

[2] Pettersson M, Crews CM. PROteolysis TArgeting Chimeras (PROTACs) - past, present and future. Drug Discov Today Technol 2019;31:15–27. 31200855 10.1016/j.ddtec.2019.01.002 PMC6578591

[3] Gu S, Cui D, Chen X, et al. PROTACs: an emerging targeting technique for protein degradation in drug discovery. BioEssays 2018;401700247. 10.1002/bies.201700247 29473971

[4] Samarasinghe KTG, Crews CM. Targeted protein degradation: a promise for undruggable proteins. Cell Chem Biol 2021;28:934–51. 34004187 10.1016/j.chembiol.2021.04.011 PMC8286327

[5] Toure M, Crews CM. Small-molecule PROTACS: new approaches to protein degradation. Angew Chem Int Ed Engl 2016;55:1966–73. 26756721 10.1002/anie.201507978

[6] Neklesa TK, Winkler JD, Crews CM. Targeted protein degradation by PROTACs. Pharmacol Ther 2017;174:138–44. 28223226 10.1016/j.pharmthera.2017.02.027

[7] Lin J, Jin J, Shen Y, et al. Emerging protein degradation strategies: expanding the scope to extracellular and membrane proteins. Theranostics 2021;11:8337–49. 34373745 10.7150/thno.62686 PMC8344007

[8] Bond MJ, Crews CM. Proteolysis targeting chimeras (PROTACs) come of age: entering the third decade of targeted protein degradation. RSC Chem Biol 2021;2:725–42. 34212149 10.1039/d1cb00011j PMC8190915

[9] Békés M, Langley DR, Crews CM. PROTAC targeted protein degraders: the past is prologue. Nat Rev Drug Discov 2022;21:181–200. 35042991 10.1038/s41573-021-00371-6 PMC8765495

[10] Lai AC, Crews CM. Induced protein degradation: an emerging drug discovery paradigm. Nat Rev Drug Discov 2017;16:101–14. 27885283 10.1038/nrd.2016.211 PMC5684876

[11] Burslem GM, Crews CM. Proteolysis-targeting chimeras as therapeutics and tools for biological discovery. Cell 2020;181:102–14. 31955850 10.1016/j.cell.2019.11.031 PMC7319047

[12] An S, Fu L. Small-molecule PROTACs: an emerging and promising approach for the development of targeted therapy drugs. EBioMedicine 2018;36:553–62. 30224312 10.1016/j.ebiom.2018.09.005 PMC6197674

[13] Troup RI, Fallan C, Baud MGJ. Current strategies for the design of PROTAC linkers: a critical review. Explor Target Antitumor Ther 2020;1:273–312. 36046485 10.37349/etat.2020.00018 PMC9400730

[14] Varshavsky A. The ubiquitin system, autophagy, and regulated protein degradation. Annu Rev Biochem 2017;86:123–28. 28654326 10.1146/annurev-biochem-061516-044859

[15] Schneider M, Radoux CJ, Hercules A, et al. The PROTACtable genome. Nat Rev Drug Discov 2021;20:789–97. 34285415 10.1038/s41573-021-00245-x

[16] Wang Y, Jiang X, Feng F, et al. Degradation of proteins by PROTACs and other strategies. Acta Pharm Sin B 2020;10:207–38. 32082969 10.1016/j.apsb.2019.08.001 PMC7016280

[17] Burslem GM, Smith BE, Lai AC, et al. The advantages of targeted protein degradation over inhibition: an RTK case study. Cell Chem Biol 2018;25:67–77 e63. 29129716 10.1016/j.chembiol.2017.09.009 PMC5831399

[18] Cromm PM, Samarasinghe KTG, Hines J, et al. Addressing kinase-independent functions of Fak via PROTAC-mediated degradation. J Am Chem Soc 2018;140:17019–26. 30444612 10.1021/jacs.8b08008

[19] Gao H, Zheng C, Du J, et al. FAK-targeting PROTAC as a chemical tool for the investigation of non-enzymatic FAK function in mice. Protein Cell 2020;11:534–9. 32451721 10.1007/s13238-020-00732-8 PMC7305269

[20] Bondeson DP, Mares A, Smith IE, et al. Catalytic in vivo protein knockdown by small-molecule PROTACs. Nat Chem Biol 2015;11:611–7. 26075522 10.1038/nchembio.1858 PMC4629852

[21] Mares A, Miah AH, Smith IED, et al. Extended pharmacodynamic responses observed upon PROTAC-mediated degradation of RIPK2. Commun Biol 2020;3:140. 32198438 10.1038/s42003-020-0868-6 PMC7083851

[22] You I, Erickson EC, Donovan KA, et al. Discovery of an AKT degrader with prolonged inhibition of downstream signaling. Cell Chem Biol 2020;27:66–73.e7.e67. 31859249 10.1016/j.chembiol.2019.11.014 PMC6980747

[23] Tomoshige S, Nomura S, Ohgane K, et al. Discovery of small molecules that induce the degradation of huntingtin. Angew Chem Int Ed Engl 2017;56:11530–3. 28703441 10.1002/anie.201706529

[24] Wang W, Zhou Q, Jiang T, et al. A novel small-molecule PROTAC selectively promotes tau clearance to improve cognitive functions in Alzheimer-like models. Theranostics 2021;11:5279–95. 33859747 10.7150/thno.55680 PMC8039949

[25] Deger JM, Gerson JE, Kayed R. The interrelationship of proteasome impairment and oligomeric intermediates in neurodegeneration. Aging Cell 2015;14:715–24. 26053162 10.1111/acel.12359 PMC4568959

[26] Cliffe R, Sang JC, Kundel F, et al. Filamentous aggregates are fragmented by the proteasome holoenzyme. Cell Rep 2019;26:2140–9.e3.e2143. 30784595 10.1016/j.celrep.2019.01.096 PMC6381791

[27] Ciechanover A, Kwon YT. Degradation of misfolded proteins in neurodegenerative diseases: therapeutic targets and strategies. Exp Mol Med 2015;47:e147–e147. 25766616 10.1038/emm.2014.117 PMC4351408

[28] Galves M, Rathi R, Prag G, et al. Ubiquitin signaling and degradation of aggregate-prone proteins. Trends Biochem Sci 2019;44:872–84. 31079890 10.1016/j.tibs.2019.04.007

[29] Thibaudeau TA, Anderson RT, Smith DM. A common mechanism of proteasome impairment by neurodegenerative disease-associated oligomers. Nat Commun 2018;9:1097. 29545515 10.1038/s41467-018-03509-0 PMC5854577

[30] Li Z, Zhu C, Ding Y, et al. ATTEC: a potential new approach to target proteinopathies. Autophagy 2020;16:185–7. 31690177 10.1080/15548627.2019.1688556 PMC6984452

[31] Izzo A, Mollo N, Nitti M, et al. Mitochondrial dysfunction in down syndrome: molecular mechanisms and therapeutic targets. Mol Med 2018;24:2. 10.1186/s10020-018-0004-y PMC6016872 30134785

[32] Takahashi D, Moriyama J Nakamura T, et al. AUTACs: cargo-specific degraders using selective autophagy. Mol Cell 2019;76:797–810.e10.e710. 31606272 10.1016/j.molcel.2019.09.009

[33] Norat P, Soldozy S, Sokolowski, et al. Mitochondrial dysfunction in neurological disorders: exploring mitochondrial transplantation. npj Regen Med 2020;5:22. 33298971 10.1038/s41536-020-00107-x PMC7683736

[34] Li H-Y, Peng Z-G. Targeting lipophagy as a potential therapeutic strategy for nonalcoholic fatty liver disease. Biochem Pharmacol 2022;197:114933. 35093393 10.1016/j.bcp.2022.114933

[35] Ercoli G, Fernandes VE, Chung WY, et al. Intracellular replication of Streptococcus pneumoniae inside splenic macrophages serves as a reservoir for septicaemia. Nat Microbiol 2018;3:600–10. 29662129 10.1038/s41564-018-0147-1 PMC6207342

[36] Deretic V. Autophagy in tuberculosis. Cold Spring Harb Perspect Med 2014;4:a018481. 25167980 10.1101/cshperspect.a018481 PMC4208715

[37] Paik S, Kim JK, Chung C, et al. Autophagy: a new strategy for host-directed therapy of tuberculosis. Virulence 2019;10:448–59. 30322337 10.1080/21505594.2018.1536598 PMC6550549

[38] Sharma V, Verma S, Seranova E, et al. Selective autophagy and xenophagy in infection and disease. Front Cell Dev Biol 2018;6:147. 30483501 10.3389/fcell.2018.00147 PMC6243101

[39] Ding Y, Fei Y, Lu B. Emerging new concepts of degrader technologies. Trends Pharmacol Sci 2020;41:464–74. 32416934 10.1016/j.tips.2020.04.005 PMC7177145

[40] Takahashi D, Arimoto H. Selective autophagy as the basis of autophagy-based degraders. Cell Chem Biol 2021;28:1061–71. 34087173 10.1016/j.chembiol.2021.05.006

[41] Alabi S, Crews C. Major advances in targeted protein degradation: PROTACs, LYTACs, and MADTACs. J Biol Chem 2021;296:100647. 33839157 10.1016/j.jbc.2021.100647 PMC8131913

[42] Pei J, Wang G, Feng L, et al. Targeting lysosomal degradation pathways: new strategies and techniques for drug discovery. J Med Chem 2021;64:3493–507. 33764774 10.1021/acs.jmedchem.0c01689

[43] Bonam SR, Wang F, Muller S. Lysosomes as a therapeutic target. Nat Rev Drug Discov 2019;18:923–48. 31477883 10.1038/s41573-019-0036-1 PMC7097195

[44] Kocak M, Ezazi Erdi S, Jorba G, et al. Targeting autophagy in disease: established and new strategies. Autophagy 2022;18:473–95. 34241570 10.1080/15548627.2021.1936359 PMC9037468

[45] Yim WW, Mizushima N. Lysosome biology in autophagy. Cell Discov 2020;6:6. 32047650 10.1038/s41421-020-0141-7 PMC7010707

[46] Banik SM, Pedram K, Wisnovsky S, et al. Lysosome-targeting chimaeras for degradation of extracellular proteins. Nature 2020;584:291–7. 32728216 10.1038/s41586-020-2545-9 PMC7727926

[47] Ahn G, Banik SM, Miller CL, et al. LYTACs that engage the asialoglycoprotein receptor for targeted protein degradation. Nat Chem Bio, 2021;17:937–46. 33767387 10.1038/s41589-021-00770-1 PMC8387313

[48] Cotton AD, Nguyen DP, Gramespacher JA, et al. Development of antibody-based PROTACs for the degradation of the cell-Surface immune checkpoint protein PD-L1. J Am Chem Soc 2021;143:593–8. 33395526 10.1021/jacs.0c10008 PMC8154509

[49] Caianiello DF, Zhang M, Ray JD, et al. Bifunctional small molecules that mediate the degradation of extracellular proteins. Nat Chem Biol 2021;17:947–53. 34413525 10.1038/s41589-021-00851-1

[50] Cassidy K, Zhao H. Redefining the scope of targeted protein degradation: translational opportunities in hijacking the autophagy-lysosome pathway. Biochemistry 2021;XXX:XXXX–XXXX. 10.1021/acs.biochem.1c00330 34569233

[51] Xie Z, Klionsky DJ. Autophagosome formation: core machinery and adaptations. Nat Cell Biol 2007;9:1102–9. 17909521 10.1038/ncb1007-1102

[52] Ohsumi Y. Historical landmarks of autophagy research. Cell Res 2014;24:9–23. 24366340 10.1038/cr.2013.169 PMC3879711

[53] Ji CH, Kim HY, Lee MJ, et al. The AUTOTAC chemical biology platform for targeted protein degradation via the autophagy-lysosome system. Nat Commun 2022;13:904. 35173167 10.1038/s41467-022-28520-4 PMC8850458

[54] Nomura D, Zoncu R, Ward C, et al. Methods and compounds for targeted antophagy. PCT International Patent WO 2019183600A1, application filed March 22 2019 (2019).

[55] Li Z, Wang C, Wang Z, et al. Allele-selective lowering of mutant HTT protein by HTT-LC3 linker compounds. Nature 2019;575:203–9. 31666698 10.1038/s41586-019-1722-1

[56] Pei J, Pan X, Wang A, et al. Developing potent LC 3-targeting AUTAC tools for protein degradation with selective autophagy. Chem Commun (Camb), 2021;57:13194–7. 10.1039/d1cc04661f 34816823

[57] Fu Y, Chen N, Wang Z, et al. Degradation of lipid droplets by chimeric autophagy-tethering compounds. Cell Res 2021. 10.1038/s41422-021-00532-7 PMC8410765 34239073

[58] Melia TJ, Lystad AH, Simonsen A. Autophagosome biogenesis: from membrane growth to closure. J Cell Biol 2020;219. 10.1083/jcb.202002085 PMC7265318 32357219

[59] Chang C, Jensen LE, Hurley JH. Autophagosome biogenesis comes out of the black box. Nat Cell Biol 2021;23:450–6. 33903736 10.1038/s41556-021-00669-y PMC8122082

[60] Mercer TJ, Gubas A, Tooze SA. A molecular perspective of mammalian autophagosome biogenesis. J Biol Chem 2018;293:5386–95. 29371398 10.1074/jbc.R117.810366 PMC5900756

[61] Yu L, Chen Y, Tooze SA. Autophagy pathway: cellular and molecular mechanisms. Autophagy 2018;14:207–15. 28933638 10.1080/15548627.2017.1378838 PMC5902171

[62] Carlsson SR, Simonsen A. Membrane dynamics in autophagosome biogenesis. J Cell Sci 2015;128:193–205. 25568151 10.1242/jcs.141036

[63] Nakatogawa H. Mechanisms governing autophagosome biogenesis. Nat Rev Mol Cell Biol 2020;21:439–58. 32372019 10.1038/s41580-020-0241-0

[64] Dikic I, Elazar Z. Mechanism and medical implications of mammalian autophagy. Nat Rev Mol Cell Biol 2018;19:349–64. 29618831 10.1038/s41580-018-0003-4

[65] Levine B, Kroemer, G. Biological functions of autophagy genes: a disease perspective. Cell 2019;176:11–42. 30633901 10.1016/j.cell.2018.09.048 PMC6347410

[66] Itakura E, Mizushima N. Characterization of autophagosome formation site by a hierarchical analysis of mammalian Atg proteins. Autophagy 2010;6:764–76. 20639694 10.4161/auto.6.6.12709 PMC3321844

[67] Hurley JH, Young LN. Mechanisms of autophagy initiation. Annu Rev Biochem 2017;86:225–44. 28301741 10.1146/annurev-biochem-061516-044820 PMC5604869

[68] Nishimura T, Tooze SA. Emerging roles of ATG proteins and membrane lipids in autophagosome formation. Cell Discov 2020;6:32. 32509328 10.1038/s41421-020-0161-3 PMC7248066

[69] Walker SA, Ktistakis NT. Autophagosome biogenesis machinery. J Mol Biol 2020;432:2449–61. 31705882 10.1016/j.jmb.2019.10.027

[70] Li L, Tong M, Fu Y, et al. Lipids and membrane-associated proteins in autophagy. Protein Cell 2020;12:520–44. 33151516 10.1007/s13238-020-00793-9 PMC8225772

[71] Noda NN. Atg2 and Atg9: intermembrane and interleaflet lipid transporters driving autophagy. Biochim Biophys Acta Mol Cell Biol Lipids 2021;1866:158956. 33932584 10.1016/j.bbalip.2021.158956

[72] Mizushima N. The ATG conjugation systems in autophagy. Curr Opin Cell Biol 2020;63:1–10. 31901645 10.1016/j.ceb.2019.12.001

[73] Johansen T, Lamark T. Selective autophagy: ATG8 family proteins, LIR motifs and cargo receptors. J Mol Biol 2020;432:80–103. 31310766 10.1016/j.jmb.2019.07.016

[74] Wesch N, Kirkin V, Rogov VV. Atg 8-family proteins—structural features and molecular interactions in autophagy and beyond. Cells 2020;9:2008. 10.3390/cells9092008 PMC7564214 32882854

[75] Lamark T, Johansen T. Mechanisms of selective autophagy. Annu Rev Cell Dev Biol 2021;37:143–69. 34152791 10.1146/annurev-cellbio-120219-035530

[76] Kirkin V, Rogov VVA. Diversity of selective autophagy receptors determines the specificity of the autophagy pathway. Mol Cell 2019;76:268–85. 31585693 10.1016/j.molcel.2019.09.005

[77] Mancias JD, Wang X, Gygi SP, et al. Quantitative proteomics identifies NCOA4 as the cargo receptor mediating ferritinophagy. Nature 2014;509:105–9. 24695223 10.1038/nature13148 PMC4180099

[78] Ma X, Lu C, Chen Y, et al. CCT2 is an aggrephagy receptor for clearance of solid protein aggregates. Cell. 10.1016/j.cell.2022.03.005 35366418

[79] Strappazzon F, Nazio F, Corrado M, et al. AMBRA1 is able to induce mitophagy via LC3 binding, regardless of PARKIN and p62/SQSTM1. Cell Death Differ 2014;22:419–32. 25215947 10.1038/cdd.2014.139 PMC4326570

[80] Di Rienzo M, Antonioli M, Fusco C, et al. Autophagy induction in atrophic muscle cells requires ULK1 activation by TRIM32 through unanchored K63-linked polyubiquitin chains. Sci Adv 2019;5:eaau8857. 31123703 10.1126/sciadv.aau8857 PMC6527439

[81] Van Humbeeck C, Cornelissen T, Hofkens H, et al. Parkin interacts with Ambra1 to induce mitophagy. J Neurosci 2011;31:10249–61. 21753002 10.1523/JNEUROSCI.1917-11.2011 PMC6623066

[82] Onishi M, Yamano K, Sato M, et al. Molecular mechanisms and physiological functions of mitophagy. EMBO J 2021;40, e104705. 33438778 10.15252/embj.2020104705 PMC7849173

[83] Khaminets A, Heinrich T, Mari M, et al. Regulation of endoplasmic reticulum turnover by selective autophagy. Nature 2015;522:354–8. 26040720 10.1038/nature14498

[84] Chu CY, Ji J, Dagda RK, et al. Cardiolipin externalization to the outer mitochondrial membrane acts as an elimination signal for mitophagy in neuronal cells. Nat Cell Biol 2013;15:1197–205. 24036476 10.1038/ncb2837 PMC3806088

[85] Sentelle RD, Senkal CE, Jiang W, et al. Ceramide targets autophagosomes to mitochondria and induces lethal mitophagy. Nat Chem Biol 2012;8:831–8. 22922758 10.1038/nchembio.1059 PMC3689583

[86] Hara-Kuge S, Fujiki Y. The peroxin Pex14p is involved in LC 3-dependent degradation of mammalian peroxisomes. Exp Cell Res 2008;314:3531–41. 10.1016/j.yexcr.2008.09.015 18848543

[87] Jiang L, Hara-Kuge S, Yamashita S-I, et al. Peroxin Pex14p is the key component for coordinated autophagic degradation of mammalian peroxisomes by direct binding to LC3-II. Genes Cells 2015;20:36–49. 25358256 10.1111/gtc.12198

[88] Nthiga TM, Shrestha BK, Lamark T, et al. CALCOCO1 is a soluble reticulophagy receptor. Autophagy 2020;16:1729–31. 32684083 10.1080/15548627.2020.1797289 PMC8386633

[89] Nthiga TM, Shrestha BK, Bruun JA, et al. Regulation of Golgi turnover by CALCOCO1-mediated selective autophagy. J Cell Biol 2021;220. 10.1083/jcb.202006128 PMC8059076 33871553

[90] Lu LQ, Tang MZ, Qi ZH, et al. Regulation of the Golgi apparatus via GOLPH 3-mediated new selective autophagy. Life Sci 2020;253:117700. 10.1016/j.lfs.2020.117700 32335164

[91] Ravenhill BJ, Boyle KB, von Muhlinen N, et al. The cargo receptor NDP52 initiates selective autophagy by recruiting the ULK complex to cytosol-invading bacteria. Mol Cell 2019;74: 320–9 e326. 30853402 10.1016/j.molcel.2019.01.041 PMC6477152

[92] Filimonenko M, Isakson P, Finley KD, et al. The selective macroautophagic degradation of aggregated proteins requires the PI3P-binding protein Alfy. Mol Cell 2010;38:265–79. 20417604 10.1016/j.molcel.2010.04.007 PMC2867245

[93] Fox LM, Kim K, Johnson CW, et al. Huntington’s disease pathogenesis is modified in vivo by Alfy/Wdfy3 and selective macroautophagy. Neuron 2010;105:813–21 e816. 10.1016/j.neuron.2019.12.003 PMC7060123 31899071

[94] Rui YN, Xu Z, Patel B, et al. Huntingtin functions as a scaffold for selective macroautophagy. Nat Cell Biol 2015;17:262–75. 25686248 10.1038/ncb3101 PMC4344873

[95] Jena KK, Kolapalli SP, Mehto S, et al. TRIM16 controls turnover of protein aggregates by modulating NRF2, ubiquitin system, and autophagy: implication for tumorigenesis. Mol Cell Oncol 2018;5:e1532251. 30525100 10.1080/23723556.2018.1532251 PMC6276851

[96] Thurston TL, Wandel MP, von Muhlinen N, et al. Galectin 8 targets damaged vesicles for autophagy to defend cells against bacterial invasion. Nature 2012;482:414–8. 22246324 10.1038/nature10744 PMC3343631

[97] Chauhan S, Kumar S, Jain A, et al. TRIMs and galectins globally cooperate and TRIM16 and galectin-3 co-direct autophagy in endomembrane damage homeostasis. Dev Cell 2016;39:13–27. 27693506 10.1016/j.devcel.2016.08.003 PMC5104201

[98] Ji CH, Kim HY, Heo AJ, et al. The N-degron pathway mediates ER-phagy. Mol Cell 2019;75:1058–72.e1059. 31375263 10.1016/j.molcel.2019.06.028

[99] Princely Abudu Y, Pankiv S, Mathai BJ, et al. NIPSNAP1 and NIPSNAP2 act as “eat me” signals for mitophagy. Dev Cell 2019;49:509–25 e512. 30982665 10.1016/j.devcel.2019.03.013 PMC12335005

[100] Jena KK, Mehto S, Kolapalli SP, et al. TRIM16 governs the biogenesis and disposal of stress-induced protein aggregates to evade cytotoxicity: implication for neurodegeneration and cancer. Autophagy 2019;15:924–6. 30806139 10.1080/15548627.2019.1586251 PMC6526826

[101] Miyakawa K, Nishi M, Ogawa M, et al. Galectin-9 restricts hepatitis B virus replication via p62/SQSTM1-mediated selective autophagy of viral core proteins. Nat Commun 2022;13:531. 35087074 10.1038/s41467-022-28171-5 PMC8795376

[102] Koyano F, Okatsu K, Kosako H, et al. Ubiquitin is phosphorylated by PINK1 to activate parkin. Nature 2014;510:162–6. 24784582 10.1038/nature13392

[103] Chen BH, Chang YJ, Lin S, et al. Hsc70/Stub1 promotes the removal of individual oxidatively stressed peroxisomes. Nat Commun 2020;11:5267. 33077711 10.1038/s41467-020-18942-3 PMC7573593

[104] Huett A, Heath RJ, Begun J, et al. The LRR and RING domain protein LRSAM1 is an E3 ligase crucial for ubiquitin-dependent autophagy of intracellular Salmonella Typhimurium. Cell Host Microbe 2012;12:778–90. 23245322 10.1016/j.chom.2012.10.019 PMC3785244

[105] Papadopoulos C, Kravic B, Meyer H. Repair or lysophagy: dealing with damaged lysosomes. J Mol Biol 2020;432:231–9. 31449799 10.1016/j.jmb.2019.08.010

[106] Zhen Y, Radulovic M, Vietri M, et al. Sealing holes in cellular membranes. The EMBO J 2021;40:e106922. 33644904 10.15252/embj.2020106922 PMC8013788

[107] Varshavsky A. N-degron and C-degron pathways of protein degradation. Proc Natl Acad Sci U S A 2019;116:358–66. 30622213 10.1073/pnas.1816596116 PMC6329975

[108] Cha-Molstad H, Sung KS, Hwang J, et al. Amino-terminal arginylation targets endoplasmic reticulum chaperone BiP for autophagy through p62 binding. Nat Cell Biol 2015;17:917–29. 26075355 10.1038/ncb3177 PMC4490096

[109] Cha-Molstad H, Lee SH, Kim JG, et al. Regulation of autophagic proteolysis by the N-recognin SQSTM1/p62 of the N-end rule pathway. Autophagy 2019;14:359–61. 10.1080/15548627.2017.1415190 PMC5902234 29261001

[110] Kimura T, Mandell M, Deretic V. Precision autophagy directed by receptor regulators - emerging examples within the TRIM family. J Cell Sci 2016;129:881–91. 26906420 10.1242/jcs.163758 PMC6518167

[111] Kimura T, Jain A, Choi SW, et al. TRIM-mediated precision autophagy targets cytoplasmic regulators of innate immunity. J Cell Biol 2015;210:973–89. 26347139 10.1083/jcb.201503023 PMC4576868

[112] Vargas JNS, Wang C, Bunker E, et al. Spatiotemporal control of ULK1 activation by NDP52 and TBK1 during selective autophagy. Mol Cell 2019;74:347–62 e346. 30853401 10.1016/j.molcel.2019.02.010 PMC6642318

[113] Thurston TL, Boyle KB, Allen M, et al. Recruitment of TBK1 to cytosol-invading Salmonella induces WIPI 2-dependent antibacterial autophagy. EMBO J 2016;35:1779–92. 10.15252/embj.201694491 PMC5010046 27370208

[114] Kroemer G, Marino G, Levine B. Autophagy and the integrated stress response. Mol Cell 2010;40:280–93. 20965422 10.1016/j.molcel.2010.09.023 PMC3127250

[115] Kirkin V. History of the selective autophagy research: how did it begin and where does it stand today? J Mol Biol 2020;432:3–27. 31082435 10.1016/j.jmb.2019.05.010 PMC6971693

[116] Abdrakhmanov A, Gogvadze V, Zhivotovsky B. To eat or to die: deciphering selective forms of autophagy. Trends Biochem Sci 2020;45:347–64. 32044127 10.1016/j.tibs.2019.11.006

[117] Zaffagnini G, Martens, S. Mechanisms of selective autophagy. J Mol Biol 2016;428:1714–24. 26876603 10.1016/j.jmb.2016.02.004 PMC4871809

[118] Eickhorst C, Licheva M, Kraft C. Scaffold proteins in bulk and selective autophagy. Prog Mol Biol Transl Sci 2020;172:15–35. 32620241 10.1016/bs.pmbts.2020.01.009

[119] Stolz A, Ernst A, Dikic I. Cargo recognition and trafficking in selective autophagy. Nat Cell Biol 2014;16:495–501. 24875736 10.1038/ncb2979

[120] Gatica D, Lahiri V, Klionsky DJ. Cargo recognition and degradation by selective autophagy. Nat Cell Biol 2018;20:233–42. 29476151 10.1038/s41556-018-0037-z PMC6028034

[121] Khaminets A, Behl C, Dikic I. Ubiquitin-dependent and independent signals in selective autophagy. Trends Cell Biol 2016;26:6–16. 26437584 10.1016/j.tcb.2015.08.010

[122] Grumati P, Dikic I. Ubiquitin signaling and autophagy. J Biol Chem 2018;293:5404–13. 29187595 10.1074/jbc.TM117.000117 PMC5900779

[123] Turco E, Fracchiolla D, Martens, S. Recruitment and activation of the ULK1/Atg1 kinase complex in selective autophagy. J Mol Biol 2020;432:123–34. 31351898 10.1016/j.jmb.2019.07.027 PMC6971721

[124] Yang Q, Zhao J, Chen D, et al. E3 ubiquitin ligases: styles, structures and functions. Mol Biomed 2021;2:23. 35006464 10.1186/s43556-021-00043-2 PMC8607428

[125] Yau R, Rape M. The increasing complexity of the ubiquitin code. Nat Cell Biol 2016;18:579–86. 27230526 10.1038/ncb3358

[126] Haakonsen DL, Rape M. Branching out: improved signaling by heterotypic ubiquitin chains. Trends Cell Biol 2019;29:704–16. 31300189 10.1016/j.tcb.2019.06.003

[127] Oh E, Akopian D, Rape M. Principles of ubiquitin-dependent signaling. Annu Rev Cell Dev Biol 2018;34:137–62. 30110556 10.1146/annurev-cellbio-100617-062802

[128] Kwon YT, Ciechanover A. The ubiquitin code in the ubiquitin-proteasome system and autophagy. Trends Biochem Sci 2017;42:873–86. 28947091 10.1016/j.tibs.2017.09.002

[129] Lamark T, Johansen T. Aggrephagy: selective disposal of protein aggregates by macroautophagy. Int J Cell Biol 2012;2012:736905. 22518139 10.1155/2012/736905 PMC3320095

[130] Yao TP. The role of ubiquitin in autophagy-dependent protein aggregate processing. Genes Cancer 2010;1:779–86. 21113398 10.1177/1947601910383277 PMC2991150

[131] Matsuda N, Sato S, Shiba K, et al. PINK1 stabilized by mitochondrial depolarization recruits Parkin to damaged mitochondria and activates latent Parkin for mitophagy. J Cell Biol 2010;189:211–21. 20404107 10.1083/jcb.200910140 PMC2856912

[132] Kane LA, Lazarou M, Fogel AI, et al. PINK1 phosphorylates ubiquitin to activate Parkin E3 ubiquitin ligase activity. J Cell Biol 2014;205:143–53. 24751536 10.1083/jcb.201402104 PMC4003245

[133] Palikaras K, Lionaki E, Tavernarakis N. Mechanisms of mitophagy in cellular homeostasis, physiology and pathology. Nat Cell Biol 2018;20:1013–22. 30154567 10.1038/s41556-018-0176-2

[134] Zachari M, Gudmundsson SR, Li Z, et al. Selective autophagy of mitochondria on a ubiquitin-endoplasmic-reticulum platform. Dev Cell 2019;50:627–43.e625. 31353311 10.1016/j.devcel.2019.06.016 PMC6739445

[135] Farré JC, Mahalingam SS, Proietto M, et al. Peroxisome biogenesis, membrane contact sites, and quality control. EMBO Rep 2019;20. 10.15252/embr.201846864 PMC6322382 30530632

[136] Polajnar M, Dietz MS, Heilemann M, et al. Expanding the host cell ubiquitylation machinery targeting cytosolic Salmonella. EMBO Rep 2017;18:1572–85. 28784601 10.15252/embr.201643851 PMC5579355

[137] Ito C, Saito Y, Nozawa T, et al. Endogenous nitrated nucleotide is a key mediator of autophagy and innate defense against bacteria. Mol Cell 2013;52:794–804. 24268578 10.1016/j.molcel.2013.10.024

[138] Yamada A, Hikichi M, Nozawa T, et al. FBXO2/SCF ubiquitin ligase complex directs xenophagy through recognizing bacterial surface glycan. EMBO Rep 2021;22:e52584. 34515398 10.15252/embr.202152584 PMC8567282

[139] Otten EG, Werner E, Crespillo-Casado A, et al. Ubiquitylation of lipopolysaccharide by RNF213 during bacterial infection. Nature 2021;594:111–6. 34012115 10.1038/s41586-021-03566-4 PMC7610904

[140] Noad J, von der Malsburg A, Pathe C, et al. LUBAC-synthesized linear ubiquitin chains restrict cytosol-invading bacteria by activating autophagy and NF-κB. Nat Microbiol 2017;2:17063. 28481331 10.1038/nmicrobiol.2017.63 PMC5576533

[141] Franco LH, Nair VR, Scharn CR, et al. The ubiquitin ligase Smurf1 functions in selective autophagy of Mycobacterium tuberculosis and anti-tuberculous host defense. Cell Host Microbe 2017;21:59–72. 28017659 10.1016/j.chom.2016.11.002 PMC5699477

[142] Manzanillo PS, Ayres JS, Watson RO, et al. The ubiquitin ligase parkin mediates resistance to intracellular pathogens. Nature 2013;501:512–6. 24005326 10.1038/nature12566 PMC3886920

[143] Cohen-Kaplan V, Livneh I, Avni N, et al. p62- and ubiquitin-dependent stress-induced autophagy of the mammalian 26S proteasome. Proc Natl Acad Sci U S A 2016;113:E7490–9. 27791183 10.1073/pnas.1615455113 PMC5127335

[144] Choi WH, Yun Y, Park S, et al. Aggresomal sequestration and STUB 1-mediated ubiquitylation during mammalian proteaphagy of inhibited proteasomes. Proc Natl Acad Sci U S A 2020;117:19190–200. 10.1073/pnas.1920327117 PMC7430983 32723828

[145] Hou P, Yang K, Jia P, et al. A novel selective autophagy receptor, CCDC50, delivers K63 polyubiquitination-activated RIG-I/MDA5 for degradation during viral infection. Cell Res 2021;31:62–79. 32612200 10.1038/s41422-020-0362-1 PMC7852694

[146] Simonsen A, Birkeland HC, Gillooly DJ, et al. Alfy, a novel FYVE-domain-containing protein associated with protein granules and autophagic membranes. J Cell Sci 2004;117:4239–51. 15292400 10.1242/jcs.01287

[147] Liu X, Li Y, Wang X, et al. The BEACH-containing protein WDR81 coordinates p62 and LC3C to promote aggrephagy. J Cell Biol 2017;216:1301–20. 28404643 10.1083/jcb.201608039 PMC5412561

[148] Yoon MJ, Choi B, Kim EJ, et al. UXT chaperone prevents proteotoxicity by acting as an autophagy adaptor for p 62-dependent aggrephagy. Nat Commun 2021;12:1955. 10.1038/s41467-021-22252-7 PMC8007730 33782410

[149] Jena KK, Kolapalli SP, Mehto S, et al. TRIM16 controls assembly and degradation of protein aggregates by modulating the p62-NRF2 axis and autophagy. EMBO J 2018;37. 10.15252/embj.201798358 PMC6138442 30143514

[150] Danieli A, Martens S. p62-mediated phase separation at the intersection of the ubiquitin-proteasome system and autophagy. J Cell Sci 2018;131. 10.1242/jcs.214304 PMC7610772 30287680

[151] Sun D, Wu R, Zheng J, et al. Polyubiquitin chain-induced p62 phase separation drives autophagic cargo segregation. Cell Res 2018;28:405–15. 29507397 10.1038/s41422-018-0017-7 PMC5939046

[152] Zaffagnini G, Savova A, Danieli A, et al. p62 filaments capture and present ubiquitinated cargos for autophagy. EMBO J 2018;37. 10.15252/embj.201798308 PMC5830917 29343546

[153] Sun D, Wu R, Li P, et al. Phase separation in regulation of aggrephagy. J Mol Biol 2018;432:160–9. 10.1016/j.jmb.2019.06.026 31260696

[154] Noda NN, Wang Z, Zhang H. Liquid-liquid phase separation in autophagy. J Cell Biol 2020;219. 10.1083/jcb.202004062 PMC7401820 32603410

[155] Wurzer B, Zaffagnini G, Feacchiolla D, et al. Oligomerization of p62 allows for selection of ubiquitinated cargo and isolation membrane during selective autophagy. Elife 2015;4:e08941. 26413874 10.7554/eLife.08941 PMC4684078

[156] Long J, Gallagher TR, Cavey JR, et al. Ubiquitin recognition by the ubiquitin-associated domain of p62 involves a novel conformational switch. J Biol Chem 2008;283:5427–40. 18083707 10.1074/jbc.M704973200

[157] Nakazawa S, Oikawa D, Ishii R, et al. Linear ubiquitination is involved in the pathogenesis of optineurin-associated amyotrophic lateral sclerosis. Nat Commun 2016;7:12547. 27552911 10.1038/ncomms12547 PMC4999505

[158] Li F, Xu D, Wang Y, et al. Structural insights into the ubiquitin recognition by OPTN (optineurin) and its regulation by TBK1-mediated phosphorylation. Autophagy 2018;14:66–79. 29394115 10.1080/15548627.2017.1391970 PMC5846504

[159] Komander D, Rape M. The ubiquitin code. Ann Rev Biochem 2012;81:203–29. 22524316 10.1146/annurev-biochem-060310-170328

[160] Grice GL, Nathan JA. The recognition of ubiquitinated proteins by the proteasome. Cell Mol Life Sci 2016;73:3497–506. 27137187 10.1007/s00018-016-2255-5 PMC4980412

[161] Kim I, Lemasters JJ. Mitochondrial degradation by autophagy (mitophagy) in GFP-LC3 transgenic hepatocytes during nutrient deprivation. Am J Physiol-Cell Physiol 2010;300:C308–17. 21106691 10.1152/ajpcell.00056.2010 PMC3043636

[162] Twig G, Elorza A, Molina AJA, et al. Fission and selective fusion govern mitochondrial segregation and elimination by autophagy. EMBO J 2008;27:433–46. 18200046 10.1038/sj.emboj.7601963 PMC2234339

[163] Papadopoulos C, Meyer H. Detection and clearance of damaged lysosomes by the endo-lysosomal damage response and lysophagy. Curr Biol 2017;27:R1330–41. 29257971 10.1016/j.cub.2017.11.012

[164] Hong MH, Weng IC, Li FY, et al. Intracellular galectins sense cytosolically exposed glycans as danger and mediate cellular responses. J Biomed Sci 2021;28:16. 33663512 10.1186/s12929-021-00713-x PMC7931364

[165] Nguyen KM, Busino L. The biology of F-box proteins: the SCF family of E3 ubiquitin ligases. Adv Exp Med Biol 2020;1217:111–22. 31898225 10.1007/978-981-15-1025-0_8

[166] Yoshida Y, Mizushima T, Tanaka K. Sugar-recognizing ubiquitin ligases: action mechanisms and physiology. Front Physiol 2019;10:104. 30837888 10.3389/fphys.2019.00104 PMC6389600

[167] Yoshida Y, Yasuda S, Fujida T, et al. Ubiquitination of exposed glycoproteins by SCF(FBXO27) directs damaged lysosomes for autophagy. Proc Natl Acad Sci U S A 2017;114:8574–9. 28743755 10.1073/pnas.1702615114 PMC5559013

[168] Liu EA, Schultz ML, Mochida C, et al. Fbxo2 mediates clearance of damaged lysosomes and modifies neurodegeneration in the Niemann-Pick C brain. JCI Insight 2020;5. 10.1172/jci.insight.136676 PMC7605537 32931479

[169] Johannes L, Jacob R, Leffler H. Galectins at a glance. J Cell Sci 2018;131. 10.1242/jcs.208884 29717004

[170] Cheng YL, Wu YW, Kuo CF, et al. Galectin-3 inhibits galectin-8/parkin-mediated ubiquitination of group A streptococcus. mBio 2017;8. 10.1128/mBio.00899-17 PMC5527311 28743815

[171] Bell SL, Lopez KL, Cox JS, et al. Galectin-8 senses phagosomal damage and recruits selective autophagy adapter TAX1BP1 to control Mycobacterium tuberculosis infection in macrophages. mBio, 2021;e0187120. 34225486 10.1128/mBio.01871-20 PMC8406326

[172] Falcon B, Noad J, McMahon H, et al. Galectin-8-mediated selective autophagy protects against seeded tau aggregation. J Biol Chem 2018;293:2438–51. 29282296 10.1074/jbc.M117.809293 PMC5818177

[173] Yoo YD, Mun SR, Ji C, et al. N-terminal arginylation generates a bimodal degron that modulates autophagic proteolysis. Proc Natl Acad Sci U S A 2018;115:E2716–24. 29507222 10.1073/pnas.1719110115 PMC5866579

[174] Cha-Molstad H, Yu JE, Feng Z, et al. p62/SQSTM1/Sequestosome-1 is an N-recognin of the N-end rule pathway which modulates autophagosome biogenesis. Nat Commun 2017;8:102. 28740232 10.1038/s41467-017-00085-7 PMC5524641

[175] Chino H, Mizushima N. ER-phagy: quality control and turnover of endoplasmic reticulum. Trends Cell Biol 2020;30:384–98. 32302550 10.1016/j.tcb.2020.02.001

[176] Smith MD, Harley ME, Kemp AJ, et al. CCPG1 is a non-canonical autophagy cargo receptor essential for ER-phagy and pancreatic ER proteostasis. Dev Cell 2018;44:217–32.e211. 29290589 10.1016/j.devcel.2017.11.024 PMC5791736

[177] Zhou Z, Liu J, Fu T, et al. Phosphorylation regulates the binding of autophagy receptors to FIP200 Claw domain for selective autophagy initiation. Nat Commun 2021;12:1570. 33692357 10.1038/s41467-021-21874-1 PMC7946963

[178] Kostenko EV, Olabisi OO, Sahay S, et al. Ccpg1, a novel scaffold protein that regulates the activity of the Rho guanine nucleotide exchange factor Dbs. Mol Cell Biol 2006;26:8964–75. 17000758 10.1128/MCB.00670-06 PMC1636807

[179] Bhaskara RM, Grumati P, Garcia-Pardo J, et al. Curvature induction and membrane remodeling by FAM134B reticulon homology domain assist selective ER-phagy. Nat Commun 2019;10:2370. 31147549 10.1038/s41467-019-10345-3 PMC6542808

[180] Forrester A, De Leonibus C, Grumati P, et al. A selective ER-phagy exerts procollagen quality control via a Calnexin-FAM134B complex. EMBO J 2019;38:e99847. 30559329 10.15252/embj.201899847 PMC6331724

[181] Zhao J, Li Z, Li J. The crystal structure of the FAM134B-GABARAP complex provides mechanistic insights into the selective binding of FAM134 to the GABARAP subfamily. FEBS Open Bio, 2021. 10.1002/2211-5463.13340 PMC8727931 34854256

[182] Grumati P, Morozzi G, Hölper S, et al. Full length RTN3 regulates turnover of tubular endoplasmic reticulum via selective autophagy. Elife 2017;6:e25555. 28617241 10.7554/eLife.25555 PMC5517149

[183] Chen YJ, Knupp J, Arunagiri A, et al. PGRMC1 acts as a size-selective cargo receptor to drive ER-phagic clearance of mutant prohormones. Nat Commun 2021;12:5991. 34645803 10.1038/s41467-021-26225-8 PMC8514460

[184] An H, Ordureau A, Paulo JA, et al. TEX264 is an endoplasmic reticulum-resident ATG8-interacting protein critical for ER remodeling during nutrient stress. Mol Cell 2019;74:891–908 e810. 31006537 10.1016/j.molcel.2019.03.034 PMC6747008

[185] Chino H, Hatta T, Natsume T, et al. Intrinsically disordered protein TEX264 mediates ER-phagy. Mol Cell 2019;74:909–21 e906. 31006538 10.1016/j.molcel.2019.03.033

[186] Fumagalli F, Noack J, Bergmann TJ, et al. Translocon component Sec62 acts in endoplasmic reticulum turnover during stress recovery. Nat Cell Biol 2016;18:1173–84. 27749824 10.1038/ncb3423

[187] Chen Q, Xiao Y, Chai P, et al. ATL3 is a tubular ER-phagy receptor for GABARAP-mediated selective autophagy. Curr Biol 2019;29:846–55 e846. 30773365 10.1016/j.cub.2019.01.041

[188] Bhujabal Z, Birgisdottir ÅB, Sjøttem E, et al. FKBP8 recruits LC3A to mediate Parkin-independent mitophagy. EMBO Rep 2017;18:947–61. 28381481 10.15252/embr.201643147 PMC5452039

[189] Li M, Jia J, Zhang X, et al. Selective binding of mitophagy receptor protein Bcl-rambo to LC3/GABARAP family proteins. Biochem Biophys Res Commun 2020;530:292–300. 32828302 10.1016/j.bbrc.2020.07.039

[190] Murakawa T, Yamaguchi O, Hashimoto A, et al. Bcl-2-like protein 13 is a mammalian Atg32 homologue that mediates mitophagy and mitochondrial fragmentation. Nat Commun 2015;6:7527. 26146385 10.1038/ncomms8527 PMC4501433

[191] Murakawa T, Okamoto K, Omiya S, et al. A mammalian mitophagy receptor, Bcl2-L-13, recruits the ULK1 complex to induce mitophagy. Cell Rep 2019;26:338–45.e336. 30625316 10.1016/j.celrep.2018.12.050 PMC6326162

[192] Liu L, Feng D, Chen G, et al. Mitochondrial outer-membrane protein FUNDC1 mediates hypoxia-induced mitophagy in mammalian cells. Nat Cell Biol 2012;14:177–85. 22267086 10.1038/ncb2422

[193] Wei Y, Chiang WC, Sumpter R, et al. Prohibitin 2 is an inner mitochondrial membrane mitophagy receptor. Cell 2017;168: 224–38.e210. 28017329 10.1016/j.cell.2016.11.042 PMC5235968

[194] Chu CT. Mechanisms of selective autophagy and mitophagy: implications for neurodegenerative diseases. Neurobiol Dis 2019;122:23–34. 30030024 10.1016/j.nbd.2018.07.015 PMC6396690

[195] Fritsch LE, Moore ME, Sarraf SA, et al. Ubiquitin and receptor-dependent mitophagy pathways and their implication in neurodegeneration. J Mol Biol 2020;432:2510–24. 31689437 10.1016/j.jmb.2019.10.015 PMC7195237

[196] Gryzik M, Srivastava A, Longhi G, et al. Expression and characterization of the ferritin binding domain of Nuclear Receptor Coactivator-4 (NCOA4). Biochim Biophys Acta Gen Subj 2017;1861:2710–6. 28754384 10.1016/j.bbagen.2017.07.015

[197] Ohshima T, Yamamoto H, Sakamaki Y, et al. NCOA4 drives ferritin phase separation to facilitate macroferritinophagy and endosomal microferritinophagy. bioRxiv 2022.2001.2031.478434, 2022, preprint: not peer reviewed. 10.1083/jcb.202203102 PMC9452830 36066504

[198] Jiang S, Heller B, Tagliabracci VS, et al. Starch binding domain-containing protein 1/genethonin 1 is a novel participant in glycogen metabolism. J Biol Chem 2010;285:34960–71. 20810658 10.1074/jbc.M110.150839 PMC2966110

[199] Jiang S, Wells CD, Roach, PJ. Starch-binding domain-containing protein 1 (Stbd1) and glycogen metabolism: identification of the Atg8 family interacting motif (AIM) in Stbd1 required for interaction with GABARAPL1. Biochem Biophys Res Commun 2011;413:420–5. 21893048 10.1016/j.bbrc.2011.08.106 PMC3411280

[200] Wyant GA, Abu-Remaileh M, Frenkel EM, et al. NUFIP1 is a ribosome receptor for starvation-induced ribophagy. Science 2018;360:751–8. 29700228 10.1126/science.aar2663 PMC6020066

[201] Mandell MA, Jain A, Arko-Mensah J, et al. TRIM proteins regulate autophagy and can target autophagic substrates by direct recognition. Dev Cell 2014;30:394–409. 25127057 10.1016/j.devcel.2014.06.013 PMC4146662

[202] Ichimura Y, Waguri S, Sou YS, et al. Phosphorylation of p62 activates the Keap1-Nrf2 pathway during selective autophagy. Mol Cell 2013;51:618–31. 24011591 10.1016/j.molcel.2013.08.003

[203] Katsuragi Y, Ichimura Y, Komatsu M. p62/SQSTM1 functions as a signaling hub and an autophagy adaptor. FEBS J 2015;282:4672–8. 26432171 10.1111/febs.13540

[204] Deosaran E, Larsen KB, Hua R, et al. NBR1 acts as an autophagy receptor for peroxisomes. J Cell Sci 2013;126:939–52. 23239026 10.1242/jcs.114819

[205] Zientara-Rytter K, Subramani S. Autophagic degradation of peroxisomes in mammals. Biochem Soc Trans 2016;44:431–40. 27068951 10.1042/BST20150268 PMC4958620

[206] Minowa-Nozawa A, Nozawa T, Okamoto-Furuta K, et al. Rab35 GTPase recruits NDP52 to autophagy targets. Embo J 2017;36:2790–807. 28848034 10.15252/embj.201796463 PMC5599792

[207] Furuya N, Kakuta S, Sumiyoshi K, et al. NDP52 interacts with mitochondrial RNA poly(A) polymerase to promote mitophagy. EMBO Rep 2018;19. 10.15252/embr.201846363 PMC6280801 30309841

[208] Korac J, Schaeffer V, Kovacevic I, et al. Ubiquitin-independent function of optineurin in autophagic clearance of protein aggregates. J Cell Sci 2013;126:580–92. 23178947 10.1242/jcs.114926 PMC3654196

[209] Lin CY, Nozawa T, Minowa-Nozawa A, et al. Autophagy receptor tollip facilitates bacterial autophagy by recruiting galectin-7 in response to group A streptococcus infection. Front Cell Infect Microbiol 2020;10, 583137. 33425778 10.3389/fcimb.2020.583137 PMC7786282

[210] Zachari M, Ganley IG. The mammalian ULK1 complex and autophagy initiation. Essays Biochem 2017;61:585–96. 29233870 10.1042/EBC20170021 PMC5869855

[211] Kumar S, Javed R, Mudd M, et al. Mammalian hybrid pre-autophagosomal structure HyPAS generates autophagosomes. Cell 2021;184:5950–69.e5922. 34741801 10.1016/j.cell.2021.10.017 PMC8616855

[212] Turco E, et al. FIP200 claw domain binding to p62 promotes autophagosome formation at ubiquitin condensates. Mol Cell 2019;74,:330–46 e311. 30853400 10.1016/j.molcel.2019.01.035 PMC6477179

[213] Turco E, Savova A, Gere F, et al. Reconstitution defines the roles of p62, NBR1 and TAX1BP1 in ubiquitin condensate formation and autophagy initiation. Nat Commun 2021;12:5212. 34471133 10.1038/s41467-021-25572-w PMC8410870

[214] Wu W, Tian W, Hu Z, et al. ULK1 translocates to mitochondria and phosphorylates FUNDC1 to regulate mitophagy. EMBO Rep 2014;15:566–75. 24671035 10.1002/embr.201438501 PMC4210082

[215] Ohnstad AE, Delgado JM, North BJ, et al. Receptor-mediated clustering of FIP200 bypasses the role of LC3 lipidation in autophagy. EMBO J 2020;39:e104948. 33226137 10.15252/embj.2020104948 PMC7737610

[216] Ktistakis NT. The dynamics of mitochondrial autophagy at the initiation stage. Biochem Soc Trans 2021;49:2199–210. 34665253 10.1042/BST20210272 PMC8589415

[217] Dalle Pezze P, Karanasios E, Kandia V, et al. ATG13 dynamics in nonselective autophagy and mitophagy: insights from live imaging studies and mathematical modeling. Autophagy 2021;17:1131–41. 32320309 10.1080/15548627.2020.1749401 PMC8143212

[218] Shi X, Chang C, Yokom AL, et al. The autophagy adaptor NDP52 and the FIP200 coiled-coil allosterically activate ULK1 complex membrane recruitment. Elife 2020;9. 10.7554/eLife.59099 PMC7447430 32773036

[219] Heo JM, Ordureau A, Paulo JA, et al. The PINK1-PARKIN mitochondrial ubiquitylation pathway drives a program of OPTN/NDP52 recruitment and TBK1 activation to promote mitophagy. Mol Cell 2015;60:7–20. 26365381 10.1016/j.molcel.2015.08.016 PMC4592482

[220] Schlütermann D, Berleth N, Deitersen J, et al. FIP200 controls the TBK1 activation threshold at SQSTM1/p 62-positive condensates. Sci Rep 2021;11:13863. 10.1038/s41598-021-92408-4 PMC8257712 34226595

[221] Richter B, Sliter DA, Herhaus L, et al. Phosphorylation of OPTN by TBK1 enhances its binding to Ub chains and promotes selective autophagy of damaged mitochondria. Proc Natl Acad Sci U S A 2016;113:4039–44. 27035970 10.1073/pnas.1523926113 PMC4839414

[222] Ma X, Helgason E, Phung QT, et al. Molecular basis of Tank-binding kinase 1 activation by transautophosphorylation. Proc Natl Acad Sci U S A 2012;109:9378–83. 22619329 10.1073/pnas.1121552109 PMC3386122

[223] Larabi A, Devos JM, Ng SL, et al. Crystal structure and mechanism of activation of TANK-binding kinase 1. Cell Rep 2013;3:734–46. 23453971 10.1016/j.celrep.2013.01.034

[224] Helgason E, Phung QT, Dueber EC. Recent insights into the complexity of Tank-binding kinase 1 signaling networks: the emerging role of cellular localization in the activation and substrate specificity of TBK1. FEBS Lett 2013;587:1230–37. 23395801 10.1016/j.febslet.2013.01.059

[225] Matsumoto G, Shimogori T, Hattori N, et al. TBK1 controls autophagosomal engulfment of polyubiquitinated mitochondria through p62/SQSTM1 phosphorylation. Hum Mol Genet 2015;24:4429–42. 25972374 10.1093/hmg/ddv179

[226] Matsumoto G, Wada K, Okuno M, et al. Serine 403 phosphorylation of p62/SQSTM1 regulates selective autophagic clearance of ubiquitinated proteins. Mol Cell 2011;44:279–89. 22017874 10.1016/j.molcel.2011.07.039

[227] Moore AS, Holzbaur EL. Dynamic recruitment and activation of ALS-associated TBK1 with its target optineurin are required for efficient mitophagy. Proc Natl Acad Sci U S A 2016;113:E3349–58. 27247382 10.1073/pnas.1523810113 PMC4914160

[228] Hatakeyama S. TRIM family proteins: roles in autophagy, immunity, and carcinogenesis. Trends Biochem Sci 2017;42:297–311. 28118948 10.1016/j.tibs.2017.01.002

[229] Di Rienzo M, Romagnoli A, Antonioli M, et al. TRIM proteins in autophagy: selective sensors in cell damage and innate immune responses. Cell Death Differ 2020;27:887–902. 31969691 10.1038/s41418-020-0495-2 PMC7206068

[230] Yamano K, Kikuchi R, Kojima W, et al. Critical role of mitochondrial ubiquitination and the OPTN-ATG9A axis in mitophagy. J Cell Biol 2020;219. 10.1083/jcb.201912144 PMC7480101 32556086

[231] Li X, Han H, Zhou MT, et al. Proteomic analysis of the human tankyrase protein interaction network reveals its role in pexophagy. Cell Rep 2017;20:737–49. 28723574 10.1016/j.celrep.2017.06.077

[232] Fracchiolla D, Sawa-Makarska J, Zens B, et al. Mechanism of cargo-directed Atg8 conjugation during selective autophagy. Elife 2016;5. 10.7554/eLife.18544 PMC5148612 27879200

[233] Martens S, Fracchiolla D. Activation and targeting of ATG8 protein lipidation. Cell Discov 2020;6:23. 32377373 10.1038/s41421-020-0155-1 PMC7198486

[234] Chang C, Young LN, Morris KL, et al. Reconstitution of cargo-induced LC3 lipidation in mammalian selective autophagy. Sci Adv 2021;7. 10.1126/sciadv.abg4922 PMC8064641 33893090

[235] Bansal M, Maharir SC, Sailasree SP, et al. Optineurin promotes autophagosome formation by recruiting the autophagy-related Atg12-5-16L1 complex to phagophores containing the Wipi2 protein. J Biol Chem 2018;293:132–47. 29133525 10.1074/jbc.M117.801944 PMC5766911

[236] Fujita N, Itoh T, Omori H, et al. The Atg16L complex specifies the site of LC3 lipidation for membrane biogenesis in autophagy. Mol Biol Cell 2008;19:2092–100. 18321988 10.1091/mbc.E07-12-1257 PMC2366860

[237] Marshall RS, Hua Z, Mali S, et al. ATG8-binding UIM proteins define a new class of autophagy adaptors and receptors. Cell 2019;177:766–81.e724. 30955882 10.1016/j.cell.2019.02.009 PMC6810650

[238] Hartmann M, Huber J, Kramer JS, et al. Demonstrating ligandability of the LC3A and LC3B adapter interface. J Med Chem 2021;64:3720–46. 33769048 10.1021/acs.jmedchem.0c01564

[239] Steffek M, Helgason E, Popovych N, et al. A multifaceted hit-finding approach reveals novel LC3 family ligands. Biochemistry 2022. 10.1021/acs.biochem.1c00682 34985287

[240] Wang Z, Zhang H. Phase separation, transition, and autophagic degradation of proteins in development and pathogenesis. Trends Cell Biol 2019;29:417–27. 30826216 10.1016/j.tcb.2019.01.008

[241] Yamasaki A, Alam JM, Noshiro D, et al. Liquidity is a critical determinant for selective autophagy of protein condensates. Mol Cell 2020;77:1163–75 e1169. 31995729 10.1016/j.molcel.2019.12.026

[242] Peng SZ, Chen XH, Chen SJ, et al. Phase separation of Nur77 mediates celastrol-induced mitophagy by promoting the liquidity of p62/SQSTM1 condensates. Nat Commun 2021;12:5989. 34645818 10.1038/s41467-021-26295-8 PMC8514450

[243] Yang Y, Willis TL, Button RW, et al. Cytoplasmic DAXX drives SQSTM1/p62 phase condensation to activate Nrf 2-mediated stress response. Nat Commun 2019;10:3759. 10.1038/s41467-019-11671-2 PMC6704147 31434890

[244] Agudo-Canalejo J, Schultz SW, Chino H, et al. Wetting regulates autophagy of phase-separated compartments and the cytosol. Nature 2021;591:142–46. 33473217 10.1038/s41586-020-2992-3

[245] Tanaka A, Cleland MM, Xu S, et al. Proteasome and p97 mediate mitophagy and degradation of mitofusins induced by Parkin. J Cell Biol 2010;191:1367–80. 21173115 10.1083/jcb.201007013 PMC3010068

[246] Di Rita A, Peschiaroli A, D’Acunzo P, et al. HUWE1 E3 ligase promotes PINK1/PARKIN-independent mitophagy by regulating AMBRA1 activation via IKKα. Nat Commun 2018;9:3755. 30217973 10.1038/s41467-018-05722-3 PMC6138665

[247] Fu M, St-Pierre P, Shankar J, et al. Regulation of mitophagy by the Gp78 E3 ubiquitin ligase. Mol Biol Cell 2013;24:1153–62. 23427266 10.1091/mbc.E12-08-0607 PMC3623636

[248] Takahashi D, Arimoto, H. Targeting selective autophagy by AUTAC degraders. Autophagy 2020;16:765–6. 31958028 10.1080/15548627.2020.1718362 PMC7138220

[249] Richard TJC, Herzog LK, Vornberger J, et al. K 63-linked ubiquitylation induces global sequestration of mitochondria. Sci Rep 2020;10:22334. 10.1038/s41598-020-78845-7 PMC7749161 33339882

[250] Mabe S, Nagamune T, Kawahara M. Detecting protein-protein interactions based on kinase-mediated growth induction of mammalian cells. Sci Rep 2014;4:6127. 25135216 10.1038/srep06127 PMC4137342

[251] Stanton BZ, Chory EJ, Crabtree GR. Chemically induced proximity in biology and medicine. Science 2018;359:eaao5902. 29590011 10.1126/science.aao5902 PMC6417506

[252] Narendra D, Kane LA, Hauser DN, et al. p62/SQSTM1 is required for Parkin-induced mitochondrial clustering but not mitophagy; VDAC1 is dispensable for both. Autophagy 2010;6:1090–106. 20890124 10.4161/auto.6.8.13426 PMC3359490

[253] Lazarou M, Jin SM, Kane LA, et al. Role of PINK1 binding to the TOM complex and alternate intracellular membranes in recruitment and activation of the E3 ligase Parkin. Dev Cell 2012;22:320–33. 22280891 10.1016/j.devcel.2011.12.014 PMC3288275

[254] D’Acunzo P, Strappazzon F, Caruana I, et al. Reversible induction of mitophagy by an optogenetic bimodular system. Nat Commun 2019;10, 1533. 30948710 10.1038/s41467-019-09487-1 PMC6449392

[255] Loos F, Xie W, Sica V, et al. Artificial tethering of LC3 or p62 to organelles is not sufficient to trigger autophagy. Cell Death Dis 2019;10:771. 31601788 10.1038/s41419-019-2011-5 PMC6787181

[256] Tatsumi T, Takayama K, Ishii S, et al. Forced lipophagy reveals that lipid droplets are required for early embryonic development in mouse. Development 2018;145. 10.1242/dev.161893 29475974

[257] Fan XY, Guo L, Chen LN, et al. Reduction of mtDNA heteroplasmy in mitochondrial replacement therapy by inducing forced mitophagy. Nat Biomed Eng 2022;6:339–50. 35437313 10.1038/s41551-022-00881-7

[258] Minami Y, Hoshino A, Higuchi Y, et al. Liver lipophagy ameliorates nonalcoholic steatohepatitis through lysosomal lipid exocytosis. bioRxiv 2022.2002.2022.481456, 2022, preprint: not peer reviewed.

[259] Cha-Molstad H, Yu JE, Lee SH, et al. Modulation of SQSTM1/p62 activity by N-terminal arginylation of the endoplasmic reticulum chaperone HSPA5/GRP78/BiP. Autophagy 2016;12:426–8. 26797053 10.1080/15548627.2015.1126047 PMC4835953

[260] Garner TP, Long J, Layfield R, et al. Impact of p62/SQSTM1 UBA domain mutations linked to paget’s disease of bone on ubiquitin recognition. Biochemistry 2011;50:4665–74. 21517082 10.1021/bi200079n

[261] Teyssou E, Takeda T, Lebon V, et al. Mutations in SQSTM1 encoding p62 in amyotrophic lateral sclerosis: genetics and neuropathology. Acta Neuropathol 2013;125:511–22. 23417734 10.1007/s00401-013-1090-0

[262] Djajadikerta A, Keshri S, Pavel M, et al. Autophagy induction as a therapeutic strategy for neurodegenerative diseases. J Mol Biol 2020;432:2799–821. 31887286 10.1016/j.jmb.2019.12.035

[263] Suresh SN, Chakravorty A, Giridharan M, et al. Pharmacological tools to modulate autophagy in neurodegenerative diseases. J Mol Biol 2020;432:2822–42. 32105729 10.1016/j.jmb.2020.02.023

[264] Shimizu Y, Yonezawa T, Sakamoto J, et al. Identification of novel inhibitors of Keap1/Nrf2 by a promising method combining protein–protein interaction-oriented library and machine learning. Sci Rep 2021;11:7420. 33795749 10.1038/s41598-021-86616-1 PMC8016952

[265] Cianfanelli V, De Zio D, Di Bartolomeo S, et al. Ambra1 at a glance. J Cell Sci 2015;128:2003–8. 26034061 10.1242/jcs.168153

[266] Liu P, Wang Y, Zhou X-M. Reconstitution of adiposome and artificial lipid droplets. FASEB J 2015;29:LB171.

[267] Huang R, Xu Y, Wan W, et al. Deacetylation of nuclear LC3 drives autophagy initiation under starvation. Mol Cell 2015;57:456–66. 25601754 10.1016/j.molcel.2014.12.013

[268] Xu C, Wang L, Fozouni P, et al. SIRT1 is downregulated by autophagy in senescence and ageing. Nat Cell Biol 2015;22:1170–9. 10.1038/s41556-020-00579-5 PMC7805578 32989246

[269] Dou Z, Xu C, Donahue G, et al. Autophagy mediates degradation of nuclear lamina. Nature 2015;527:105–9. 26524528 10.1038/nature15548 PMC4824414

[270] Reggiori F, Monastyrska I, Verheije MH, et al. Coronaviruses Hijack the LC3-I-positive EDEMosomes, ER-derived vesicles exporting short-lived ERAD regulators, for replication. Cell Host Microbe 2010;7:500–8. 20542253 10.1016/j.chom.2010.05.013 PMC7103375

[271] Leidal AM, Huang HH, Marsh T, et al. The LC3-conjugation machinery specifies the loading of RNA-binding proteins into extracellular vesicles. Nat Cell Biol 2020;22:187–99. 31932738 10.1038/s41556-019-0450-y PMC7007875

[272] Zhang H, An P, Fei Y, et al. Modeling the degradation effects of autophagosome tethering compounds. Neurosci Bull 2021;37:255–60. 32895897 10.1007/s12264-020-00574-8 PMC7870735

[273] Hyslop LA, Blakeley P, Craven L, et al. Towards clinical application of pronuclear transfer to prevent mitochondrial DNA disease. Nature 2016;534:383–6. 27281217 10.1038/nature18303 PMC5131843

[274] Kang E, Wu J, Gutierrez NM, et al. Mitochondrial replacement in human oocytes carrying pathogenic mitochondrial DNA mutations. Nature 2016;540:270–5. 27919073 10.1038/nature20592

[275] Greenfield A, Braude P, Flinter F, et al. Assisted reproductive technologies to prevent human mitochondrial disease transmission. Nat Biotechnol 2017;35:1059–68. 29121011 10.1038/nbt.3997

[276] Coll-Martínez B, Delgado A, Crosas B. The potential of proteolytic chimeras as pharmacological tools and therapeutic agents. Molecules 2020;25. 10.3390/molecules25245956 PMC7766482 33339292

[277] Dong G, Ding Y, He S, et al. Molecular glues for targeted protein degradation: from serendipity to rational discovery. J Med Chem 2021;64:10606–20. 34319094 10.1021/acs.jmedchem.1c00895

[278] Geiger TM, Schäfer SC, Dreizler JK, et al. Clues to molecular glues. Curr Res Chem Biol 2022;2:100018.

[279] Schreiber SL. The rise of molecular glues. Cell 2021;184:3–9. 33417864 10.1016/j.cell.2020.12.020

[280] Mayor-Ruiz C, Bauer S, Brand M, et al. Rational discovery of molecular glue degraders via scalable chemical profiling. Nat Chem Biol 2021;16:1199–207. 10.1038/s41589-020-0594-x PMC7116640 32747809

[281] Ishida T, Ciulli A. E3 ligase ligands for PROTACs: how they were found and how to discover new ones. SLAS Discov 2021;26:484–502. 33143537 10.1177/2472555220965528 PMC8013866

[282] Fisher SL, Phillips AJ. Targeted protein degradation and the enzymology of degraders. Curr Opin Chem Biol 2018;44:47–55. 29885948 10.1016/j.cbpa.2018.05.004

[283] Rambacher KM, Calabrese MF, Yamaguchi M. Perspectives on the development of first-in-class protein degraders. Future Med Chem 2021;13:1203–26. 34015962 10.4155/fmc-2021-0033

[284] Casement R, Bond A, Craigon C, et al. Mechanistic and structural features of PROTAC ternary complexes. Methods Mol Biol 2021;2365:79–113. 34432240 10.1007/978-1-0716-1665-9_5

[285] Bricelj A, Steinebach C, Kuchta R, et al. E3 ligase ligands in successful PROTACs: an overview of syntheses and linker attachment points. Front Chem 2021;9:707317. 34291038 10.3389/fchem.2021.707317 PMC8287636

[286] Riching KM, Mahan S, Corona CR, et al. Quantitative live-cell kinetic degradation and mechanistic profiling of PROTAC mode of action. ACS Chem Biol 2018;13:2758–70. 30137962 10.1021/acschembio.8b00692

[287] Nabet B, Roberts JM, Buckley DL, et al. The dTAG system for immediate and target-specific protein degradation. Nat Chem Biol 2018;14:431–41. 29581585 10.1038/s41589-018-0021-8 PMC6295913

[288] Nabet B, Ferguson FM, Seong BKA, et al. Rapid and direct control of target protein levels with VHL-recruiting dTAG molecules. Nat Commun 2020;11:4687. 32948771 10.1038/s41467-020-18377-w PMC7501296

[289] Buckley DL, Raina K, Darricarrere N, et al. HaloPROTACS: use of small molecule PROTACs to induce degradation of HaloTag fusion proteins. ACS Chem Biol 2015;10:1831–7. 26070106 10.1021/acschembio.5b00442 PMC4629848

[290] Menzies FM, Fleming A, Rubinsztein DC. Compromised autophagy and neurodegenerative diseases. Nat Rev Neurosci 2015;16:345–57. 25991442 10.1038/nrn3961

[291] Ren H, Wang G. Autophagy and lysosome storage disorders. Adv Exp Med Biol 2020;1207:87–102. 32671740 10.1007/978-981-15-4272-5_5

[292] Lieberman AP, Puertollano R, Raben N, et al. Autophagy in lysosomal storage disorders. Autophagy 2012;8:719–30. 22647656 10.4161/auto.19469 PMC3378416

[293] Banerjee C, Mehra D, Song D, et al. ULK1 forms distinct oligomeric states and nanoscopic morphologies during autophagy initiation. bioRxiv 2020. 10.1126/sciadv.adh4094 PMC10541014 37774021

[294] Sternicki LM, Nonomiya J, Liu M, et al. Native mass spectrometry for the study of PROTAC GNE-987-containing ternary complexes. ChemMedChem 2021;16:2206–10. 33792163 10.1002/cmdc.202100113 PMC8359942

[295] Mathieu C, Pappu RV, Taylor JP. Beyond aggregation: pathological phase transitions in neurodegenerative disease. Science 2020;370:56–60. 33004511 10.1126/science.abb8032 PMC8359821

[296] Ray S, Singh N, Kumar R, et al. α-Synuclein aggregation nucleates through liquid–liquid phase separation. Nat Chem 2020;12:705–16. 32514159 10.1038/s41557-020-0465-9

[297] Zbinden A, Pérez-Berlanga M, De Rossi, P, et al. Phase separation and neurodegenerative diseases: a disturbance in the force. Dev Cell 2020;55:45–68. 33049211 10.1016/j.devcel.2020.09.014

